# Reducing the computational footprint for real-time BCPNN learning

**DOI:** 10.3389/fnins.2015.00002

**Published:** 2015-01-22

**Authors:** Bernhard Vogginger, René Schüffny, Anders Lansner, Love Cederström, Johannes Partzsch, Sebastian Höppner

**Affiliations:** ^1^Department of Electrical Engineering and Information Technology, Technische Universität DresdenGermany; ^2^Department of Computational Biology, School of Computer Science and Communication, Royal Institute of Technology (KTH)Stockholm, Sweden; ^3^Department of Numerical Analysis and Computer Science, Stockholm UniversityStockholm, Sweden

**Keywords:** Bayesian confidence propagation neural network (BCPNN), Hebbian learning, synaptic plasticity, event-driven simulation, spiking neural networks, look-up tables, fixed-point accuracy, digital neuromorphic hardware

## Abstract

The implementation of synaptic plasticity in neural simulation or neuromorphic hardware is usually very resource-intensive, often requiring a compromise between efficiency and flexibility. A versatile, but computationally-expensive plasticity mechanism is provided by the Bayesian Confidence Propagation Neural Network (BCPNN) paradigm. Building upon Bayesian statistics, and having clear links to biological plasticity processes, the BCPNN learning rule has been applied in many fields, ranging from data classification, associative memory, reward-based learning, probabilistic inference to cortical attractor memory networks. In the spike-based version of this learning rule the pre-, postsynaptic and coincident activity is traced in three low-pass-filtering stages, requiring a total of eight state variables, whose dynamics are typically simulated with the fixed step size Euler method. We derive analytic solutions allowing an efficient event-driven implementation of this learning rule. Further speedup is achieved by first rewriting the model which reduces the number of basic arithmetic operations per update to one half, and second by using look-up tables for the frequently calculated exponential decay. Ultimately, in a typical use case, the simulation using our approach is more than one order of magnitude faster than with the fixed step size Euler method. Aiming for a small memory footprint per BCPNN synapse, we also evaluate the use of fixed-point numbers for the state variables, and assess the number of bits required to achieve same or better accuracy than with the conventional explicit Euler method. All of this will allow a real-time simulation of a reduced cortex model based on BCPNN in high performance computing. More important, with the analytic solution at hand and due to the reduced memory bandwidth, the learning rule can be efficiently implemented in dedicated or existing digital neuromorphic hardware.

## 1. Introduction

Bayesian Confidence Propagation Neural Networks (BCPNNs) realize Bayesian statistics with spiking or non-spiking neural networks. They can be used to build powerful associative memories (Sandberg et al., 2000; Meli and Lansner, 2013) and data classifiers, with applications ranging from data mining (Bate et al., 1998; Lindquist et al., 2000) to olfaction modeling (Kaplan and Lansner, 2014). The underlying Bayesian learning rule has clear links to biological synaptic plasticity processes (Tully et al., 2014), cortical associative memory (Lansner, 2009), reinforcement learning (Johansson et al., 2003), and action selection (Berthet et al., 2012). Furthermore, BCPNNs have been used to model phenomena like synaptic working memory (Sandberg et al., 2003), word-list learning in humans (Lansner et al., 2013) and memory consolidation (Fiebig and Lansner, 2014), making it a promising paradigm for information processing in the brain, while retaining a level of abstraction suitable for efficient technical implementation. Models using more detailed spiking attractor networks with the same structure have provided non-trivial explanations for memory retrieval and other basic cognitive phenomena like e.g., attentional blink (Lundqvist et al., 2010, 2011; Silverstein and Lansner, 2011; Lundqvist et al., 2013).

The performance of BCPNNs, for example in memory tasks, scales well with network size, making them extraordinarily powerful for large networks (Johansson et al., 2001). Therefore, massively parallel simulations of these networks (29 million spiking units, 295 billion plastic connections) have been realized on supercomputers (Benjaminsson and Lansner, 2011). These showed that BCPNN implementations are bounded by computation (Johansson and Lansner, 2007). To alleviate this limit, conceptual work on implementations in neuromorphic hardware has been performed (Johansson and Lansner, 2004; Farahini et al., 2014; Lansner et al., 2014).

In this paper, we pave the way for an efficient implementation of BCPNN in digital neuromorphic hardware by reducing both its computational and memory footprint. Existing software models apply fixed step size numerical integration methods for solving the BCPNN dynamics. Although easy to implement, this clock-driven simulation approach has two major drawbacks: First, there is a relatively high base cost for calculating the updates of all state variables at every time step, irrespective of the spiking activity in the network. Second, the states have to be read from and written back to memory at every simulation step, which is especially expensive for custom hardware implementations where the states are stored in an external memory. As suggested in recent work (Lansner et al., 2014), we tackle these issues by moving to an event-driven simulation scheme, which we systematically optimize for minimal number of calculations to achieve a reduction of the computational load by an order of magnitude. This efficiency gain of the event-driven paradigm is mainly due to the sparse activity in BCPNNs, which is retained irrespective of network size. Employing pre-calculated look-up tables for the frequent calculation of the exponential function, we further minimize the computational cost per event-driven update. By using an analytical solution of the model equations, the numerical accuracy of the simulation is increased compared to conventional simulation techniques with fixed step size (Henker et al., 2012). We show how this accuracy overhead could be utilized for significantly reducing the required memory and memory bandwidth in a potential hardware implementation by using fixed point operands with fewer bits than in a floating point representation.

While we performed our equation optimizations specifically for the BCPNN model, they are not restricted to it. As BCPNNs rely on dynamic equations that are common in neuroscientific modeling, our approach can be easily adopted to other models. It shows how to efficiently calculate single neuronal traces and correlation measures for synaptic plasticity, increasing the energy efficiency of digital implementations, either on standard computers or on specialized hardware, on an algorithmic level, complementing analog approaches for increasing the energy efficiency of neuromorphic computation (Hasler and Marr, 2013).

## 2. Materials and methods

### 2.1. Bayesian confidence propagation neural networks

In BCPNNs (Lansner and Ekeberg, 1989; Lansner and Holst, 1996) the synaptic weights between network units are calculated in a Hebbian fashion by applying Bayes' rule on the past activity of the units giving a measure of the co-activation of the units. In a similar manner each unit's bias is calculated from its past activity, representing its *a priori* probability to be active. Often, the activity of the units is represented by stochastic spike events, which are generated according to each unit's recent input and own activity. Typically, in a *training* phase these correlation and activation statistics are collected, which are then used in the subsequent *test* phase to perform inference, i.e., to determine the *a posteriori* activity of some units as a response to other units' recent activity. While the concept of BCPNN was originally developed for series of discrete samples, a time-continuous spike-based version has been developed recently, which we describe in Section 2.1.1 and whose efficient simulation is the main subject of this article. In Section 2.1.2, we present an application of this spike-based BCPNN learning rule in a modular network that constitutes a reduced full-scale model of the cortex.

#### 2.1.1. Spike-based BCPNN

**Figure 1:**
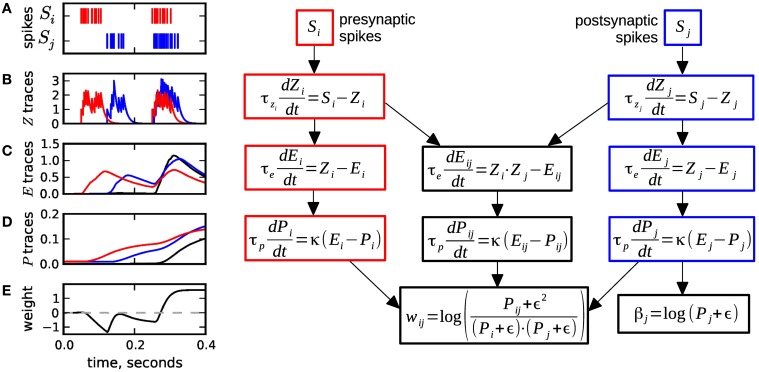
**Equations and sample traces of the spike-based BCPNN learning rule. (A)** Presynaptic (red) *S_i_* and postsynaptic (blue) *S_j_* spike trains serve as input to a BCPNN synapse. **(B)** The input spike trains are low pass filtered into the *Z* traces with time constants τ*_z_i__*,τ*_z_j__*. **(C)** *E* traces compute the low-pass filter of *Z* traces with τ_*e*_. The *E_ij_* variable (black) tracks coincident pre- and postsynaptic activity. **(D)** *E* traces are passed on to the *P* traces and low-pass filtered with τ_*p*_. **(E)** The *P* traces are used to compute the postsynaptic bias β_*j*_ and the synaptic weight *w_ij_*, which can vary between positive and negative values. Usually, the dynamics get slower from **B** to **D:** τ*_z_i__*, τ*_z_j__* ≤ τ_*e*_ ≤ τ_*p*_. Figure redrawn from Tully et al. (2014).

Spike-based BCPNN (Wahlgren and Lansner, 2001; Tully et al., 2014) is implemented by a set of local synaptic state variables that keep track of presynaptic, postsynaptic, and synaptic (i.e., correlated) activity over three different time scales, by passing spiking activity over three low pass filters, see Figure 1. Here and throughout this paper the three sites (pre-, postsynaptic and synaptic) are denoted by indices *i*, *j*, and *ij*, respectively. In the first processing stage, the pre- and postsynaptic spiking activity represented by spike trains *S_i_* (resp. *S_j_*) is low pass filtered into the *Z_i_* and *Z_j_* traces (Figure 1B), with time constants τ*_z_i__* and τ*_z_j__* in a range of 5 ms to 100 ms, which corresponds to typical synaptic decay time constants for various receptor types.

In the second stage, the *Z* traces are passed on to the *E* or eligibility traces and low pass filtered with time constant τ_*e*_. Here, a separate trace *E_ij_* is introduced to filter the coincident activity of the Z-traces, see Figure 1C. The *E* traces typically have slower dynamics than the *Z* traces (τ_*e*_ ≈ 20 − 1000 ms), and can be motivated to provide a mechanism for delayed reward learning (cf. Tully et al., 2014).

The *E* traces in turn are low pass filtered into the *P* traces (Figure 1D). These tertiary traces have the slowest dynamics with time constant τ_*p*_ ranging from 1 s to several 100 s, even higher values are possible. The *P* traces correspond to the probabilities of the units being active or co-active in the original non-spiking BCPNN formulation (Lansner and Holst, 1996). In a final step the *P* traces are used to compute the synaptic weight *w_ij_* and the postsynaptic bias β_*j*_ (Figure 1E). The formulas for *w_ij_* and β_*j*_ contain the parameter ϵ, which originates from a minimum spiking activity assumed for the pre- and postsynaptic units (cf. Tully et al., 2014), and which has the side effect to avoid division by zero in the weight formula.

The global parameter κ in the dynamics of *P* traces can take any non-negative value and controls the learning, i.e., it determines how strong recent correlations are stored. When the learning rate κ equals zero, there is no learning, as the *P* traces do not change at all, and thus neither do the synaptic weight *w_ij_* and the postsynaptic bias β_*j*_. We assume that κ only undergoes discrete and seldom changes, mostly when learning is switched on or off. Hence, while κ is constant and non-zero, the dynamics of the *P* traces can be expressed with a modified time constant τ^*^_p_:

(1) $$\tau_{p}^{*}\frac{dP}{dt}=E-P,\;\tau_{p}^{*}=\frac{\tau_{p}}{\kappa }$$

We refer to Tully et al. (2014) for establishing the link between the spike-based and the probabilistic BCPNN learning rule, as well as for details on the biological equivalents of the processing stages. Also, note that in some cases the second low pass filter is not actually used, so that the *Z* traces are directly passed to the *P* traces.

#### 2.1.2. Reduced modular model of the cortex

As an application of the spike-based BCPNN we consider a modular abstract network model, motivated by the columnar structure of the cortex, that was already presented in Lansner et al. (2014). One assumption is that the smallest functional units in the mammalian cortex are not single neurons but so-called minicolumns. A minicolumn is formed by a local population of some hundred neurons with enhanced recurrent connectivity and similar receptive fields, so that these neurons are assumed to have quite correlated output. An example would be a minicolumn encoding a certain orientation during processing in primary visual cortex.

In the order of 100 minicolumns are aggregated in a larger columnar structure, the cortical hypercolumn, which contains in the order of 10,000 neurons. Within a hypercolumn the minincolumns compete in a soft winner-take all (soft-WTA) fashion through feedback inhibition, so that most of the time only one minicolumn shows high firing activity while the others are mostly silent. Minicolumns can be viewed to encode a discrete value of an attribute specific to each hypercolumn.

In our reduced, abstract model, each minicolumn is represented by one stochastically spiking minicolumn unit (MCU). Only connections outside a hypercolumn are implemented: The internal connections between neurons of a minicolumn are hidden within the MCU, while the competitive feedback inhibition of the 100 MCUs within a hypercolumn unit (HCU) is hardwired by means of a normalization of activity per HCU (cf. Equation 5 below). In turn, for the implementation of the incoming long-range synaptic connections, which on the neuron level typically make up half of the between 10^3^ and 10^4^ incoming connections in total, we assume that each MCU propagates its spikes to 10,000 other MCUs, and has appropriately as many incoming connections. These connections are *patchy* in the sense that each MCU projects onto 100 hypercolumns and delivers spikes to all 100 MCUs of each target HCU. The connection scheme is motivated as follows: Long-range connections are provided by large layer 5 pyramidal cells, which make up around 10% of a minicolumn. Each of those cells forms synaptic connections to clusters of far away neurons in horizontal direction. The diameter of these clusters approximately corresponds to the dimension of a hypercolumn. In real cortex, each of the large pyramidal cells generates around 10 of these *patches* (Houzel et al., 1994; Binzegger et al., 2007), which motivates the 100 target HCUs per MCU, assuming that one MCU comprises one hundred neurons. All of these connections between MCUs are subject to the spike-based BCPNN learning equations of Figure 1.

**Figure 2:**
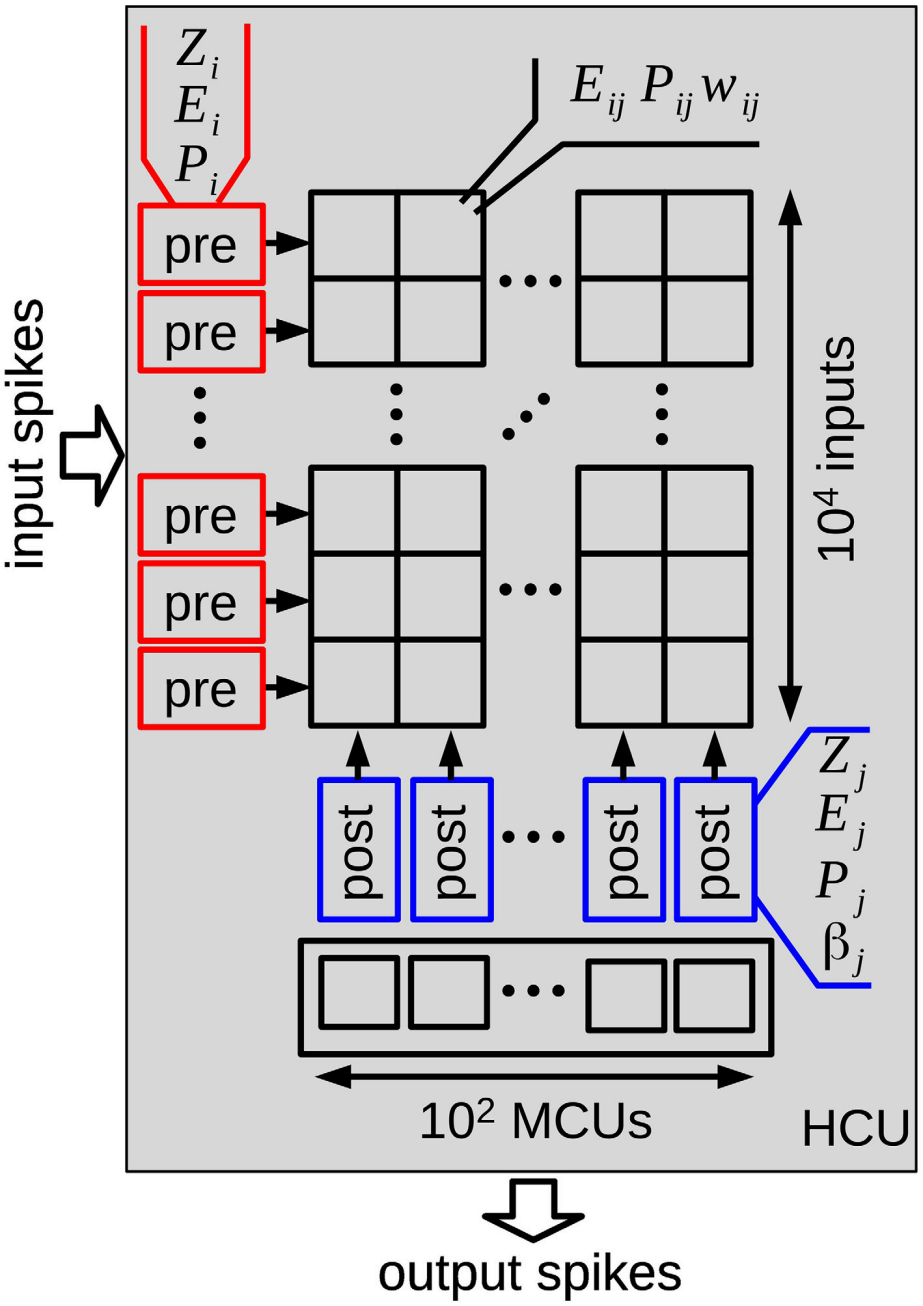
**Structure of one hypercolumn unit (HCU) of the reduced cortex model**. Each HCU contains 100 stochastic minicolumn units (MCUs) that compete in a winner-take-all fashion. Input spikes from 10,000 MCUs of other HCUs connect via 1 million BCPNN synapses to all 100 MCUs of the HCU. Each MCU sends its output spikes to 100 other HCUs. In order to store all *Z*,*E*,*P* traces and synaptic weights of the HCU, more than 12 MB memory is required when using floating point numbers with single-precision.

At a higher level, HCUs represent independent network modules between which spikes are transmitted. Each HCU consists of 100 MCUs and 1 million plastic synapses organized in an array with 10^4^ inputs and 100 outputs, as illustrated in Figure 2. The pre- and postsynaptic states of the BCPNN model can therefore be implemented at the margin of the array, while the synaptic traces *E_ij_*,*P_ij_*, and *w_ij_* form the array, thus representing the largest amount of state variables. The minicolumn units integrate the incoming spiking activity, which is then turned into a spiking probability of each unit. In particular, presynaptic input leads to a synaptic current *s*_syn,j_ (Equation 2), which together with the bias β_*j*_ and a specific external input *I_j_* sums up to the support value *s_j_* for each minicolum unit *j* in Equation (3):

(2) $$\tau_{z_{i}}\frac{ds_{\text{syn},\text{j}}(t)}{dt}=\sum_{i}w_{ij}(t)S_{i}(t)-s_{\text{syn},\text{j}}(t)$$

(3) $$s_{j}(t)=\beta_{j}(t)+s_{\text{syn},\text{j}}(t)+I_{j}(t)\;.$$

The low-pass filtered version of Equation (3) gives the “membrane potential” *m_j_* of each MCU:

(4) $$\tau_{m}\frac{dm_{j}(t)}{dt}=s_{j}(t)-m_{j}(t)\;,$$

where τ_*m*_ is the membrane time constant in the order of 10 ms. In other words, the MCUs are leaky-integrators (Equation 4) with three different input currents (Equation 3): The bias β_j_(*t*) represents the *prior* contribution to the unit's activation irrespective of the current synaptic input, determined by the past spiking activity of the unit itself via the postsynaptic traces (*Z_j_*, *E_j_*, *P_j_*, cf. Figure 1). The synaptic input is implemented as an exponentially decaying synaptic current *s*_syn,j_(*t*) (Equation 2), which - at a presynaptic spike of input *i* - is increased by synaptic weight *w_ij_*(*t*) learned according to the Equations in Figure 1. Last, the external input *I_j_*(*t*) allows a specific stimulation of single units.

All *M* MCUs of a hypercolumn unit are organized as a probabilistic soft-WTA circuit. The activation *o_j_* of each unit is computed as:

(5) $$o_{j}=\{\begin{array}{ll} \frac{{\text{e}}^{\gamma_{m}m_{j}}}{\sum_{k\;=\;1}^{M}e^{\gamma_{m}m_{k}}}, & \text{if}\sum_{k\;=\;1}^{m}e^{\gamma_{m}m_{k}}>1\\ {\text{e}}^{\gamma_{m}m_{j}} & \text{otherwise} \end{array},$$

The gain factor γ_*m*_ controls the strength of the soft-WTA filtering process, the higher γ_*m*_ the higher the activation-ratio between the winning unit and the remaining units. The normalization in Equation (5) ensures that on average not more than 1 MCU is active at the same time.

The activation *o_j_* then translates into the instantaneous Poisson firing rate *r_j_* for each unit:

(6) $$r_{j}(t)=o_{j}(t)\cdot r_{\text{max},\text{HCU}}$$

where *r*_max,HCU_ is the maximum firing rate per HCU. The average spiking frequency in mammalian cortex is quite sparse, with an average spike rate on the order of 0.1 Hz (Lennie, 2003). In our full scale HCU with 100 MCUs the average activity level would be around 1 Hz (thus *r*_max,HCU_ = 100 HZ), and the difference is explained by the fact that one MCU represents around 10 layer 5 pyramidal cells.

### 2.2. Simulation strategies

#### 2.2.1. Fixed step size simulation

The typical approach for the simulation of spiking neural networks is simulation with fixed step size, where all states are synchronously updated at every tick of a clock (Brette et al., 2007; Henker et al., 2012). Usually, in such time-driven simulation, one uses numerical integration methods like Euler or Runge-Kutta to advance the state by one time step *dt*.

For our reference fixed step size simulation we follow Lansner et al. (2014) and use the explicit Euler method for the numerical integration with a rather long time step of *dt* = 1 ms. As the MCUs are stochastic, the instantaneous firing rate *r_j_* (Equation 6) is transformed into a firing probability per time step, which is then compared to a uniform random number between 0 and 1 to generate spikes. The 1 ms time step is also used in state-of-the-art real-time digital neuromorphic systems like the SpiNNaker (Furber et al., 2014) and the Synapse hardware (Merolla et al., 2014). For completeness, we also present results with 0.1 ms step size, which is commonly used for the simulation of spiking neural networks.

#### 2.2.2. Event-driven simulation

In Sections 2.3.1 and 2.3.3 we provide analytical solutions for the spike-based BCPNN model. For those simulations we mix the time-driven and event-driven approach: We restrict spike times to multiples of the simulation time step *dt*. The stochastic MCUs (Equations 2–6) are evaluated as for the time-driven approach, which requires that also the β_*j*_ is computed at every time step. In contrast, the states of the BCPNN synapses (Figure 1) are only updated at the occurrence of a pre- or postsynaptic event.

### 2.3. Analytical solutions of spike-based BCPNN

The simulation of spike-based BCPNN with a fixed step size method is cost-intensive and requires very frequent read and write of the state variables from and to memory. Therefore, we first provide the rather straightforward analytical solution of the BCPNN equations in Section 2.3.1, allowing an exact event-driven simulation scheme. As intermediate step, we rewrite the BCPNN dynamics as a spike response model in Section 2.3.2, which then provides the basis for a second analytical solution with reduced number of operations (Section 2.3.3). Although not employed in the experiments in this article, discrete changes of the learning rate κ must be considered for the completeness of the two analytical solutions, which is done in Appendix A3.

#### 2.3.1. BCPNN solution: analytical I

For the event-driven simulation of BCPNN, the update of the state variables is only triggered by events (usually pre- or postsynaptic spikes). For each state variable one requires the time of its last update *t*^last^, in contrast to the time-driven simulation, where all states correspond to the same global time. Event-driven simulations are especially efficient if the update of the states from *t*^last^ to the current time *t* can be solved analytically. Without further derivation, we give the analytic solution to advance the *Z*, *E* and *P* traces by Δ*t* = *t* − *t*^last^ from time *t*^last^ to *t*, provided that there is no spike between *t*^last^ and *t*. For the presynaptic traces the solutions are

(7) $$Z_{i}(t)=Z_{i}(t^{\text{last}})\cdot {\text{e}}^{-\frac{\Delta t}{\tau_{z_{i}}}}{+\text{S}}_{i}(t)$$

(8) $$E_{i}(t)=E_{i}(t^{\text{last}})\cdot {\text{e}}^{-\frac{\Delta t}{\tau_{\text{e}}}}+Z_{i}(t^{\text{last}})a_{i}\left({\text{e}}^{-\frac{\Delta t}{\tau_{z_{i}}}}-{\text{e}}^{-\frac{\Delta t}{\tau_{\text{e}}}}\right)$$

(9) $$\begin{array}{l} P_{i}(t)=P_{i}(t^{\text{last}})\cdot {\text{e}}^{-\frac{\Delta t}{\tau_{p}^{*}}}+a_{i}b_{i}\left({\text{e}}^{-\frac{\Delta t}{\tau_{z_{i}}}}-{\text{e}}^{-\frac{\Delta t}{\tau_{p}^{*}}}\right)Z_{i}(t^{\text{last}})\\ \;+\left(E_{i}(t^{\text{last}})-a_{i}Z_{i}(t^{\text{last}})\right)c\left({\text{e}}^{-\frac{\Delta t}{\tau_{\text{e}}}}-{\text{e}}^{-\frac{\Delta t}{\tau_{p}^{*}}}\right)\;, \end{array}$$

with the following coefficients used for brevity:

(10) $$a_{i}=\frac{\tau_{z_{i}}}{\tau_{z_{i}}-\tau_{e}}\;,\;b_{i}=\frac{\tau_{z_{i}}}{\tau_{z_{i}}-\tau_{p}^{*}}\;,\;c=\frac{\tau_{e}}{\tau_{e}-\tau_{p}^{*}}\;.$$

In Equation (7) *S_i_* describes the presynaptic spike train taking value 1 at the spike time *t^f^_i_* and value 0 otherwise, formally

(11) $$S_{i}(t)\;=\sum_{t_{i}^{f}}\delta (t-t_{i}^{\;f})\;,$$

where δ(·) denotes a Dirac pulse. We note that Equation (8) is only valid when τ*_z_i__* ≠ τ_*e*_, Equation (9) furthermore requires that τ^*^_p_ is different from both τ*_z_i__* and τ_*e*_. For the sake of simplicity we restrict ourselves within this article to time constants fulfilling this condition, but give the solution for the other cases in Appendix A.

The update formulas for the postsynaptic traces *Z_j_*, *E_j_*, and *P_j_* can be obtained by replacing indices *i* by *j* in the presynaptic update formulas.

Accordingly, the update of the synaptic traces *E_ij_* and *P_ij_* is given by:

(12) $$E_{ij}(t)\text{​ }=E_{ij}(t^{\text{last}})\text{​}\cdot \text{​}{\text{e}}^{-\frac{\Delta t}{\tau_{e}}}+Z_{i}(t^{\text{last}})Z_{j}(t^{\text{last}})a_{ij}\left({\text{e}}^{-\frac{\Delta t}{\tau_{z_{ij}}}}\text{​}-{\text{e}}^{-\frac{\Delta t}{\tau_{e}}}\right)$$

(13) $$\begin{array}{l} P_{ij}(t)=P_{ij}(t^{\text{last}})\cdot {\text{e}}^{-\frac{\Delta t}{\tau_{p}^{*}}}+a_{ij}b_{ij}\left({\text{e}}^{-\frac{\Delta t}{\tau_{z_{ij}}}}-{\text{e}}^{-\frac{\Delta t}{\tau_{p}^{*}}}\right)Z_{i}(t^{\text{last}})Z_{j}(t^{\text{last}})\\ \;+\left(E_{ij}(t^{\text{last}})-a_{ij}Z_{i}(t^{\text{last}})Z_{j}(t^{\text{last}})\right)c\left({\text{e}}^{-\frac{\Delta t}{\tau_{e}}}-{\text{e}}^{-\frac{\Delta t}{\tau_{p}^{*}}}\right), \end{array}$$

with shortcuts

(14) $$\tau_{z_{ij}}={\left(\frac{1}{\tau_{z_{i}}}+\frac{1}{\tau_{z_{j}}}\right)}^{-1}\;,a_{ij}=\frac{\tau_{z_{ij}}}{\tau_{z_{ij}}-\tau_{e}}\;,b_{ij}=\frac{\tau_{z_{ij}}}{\tau_{z_{ij}}-\tau_{p}^{*}}\;.$$

Note that, on purpose, Equations (9, 13) were not further simplified to ease the comparison with the spike response model formulation of the BCPNN model in the next section. Again, we restrict ourselves to parameter sets where none of the involved time constants (τ*_z_ij__*, τ_*e*_ and τ^*^_p_) are equal. Note, however, that τ*_z_i__* and τ*_z_j__* may be equal.

The analytical solution of the BCPNN equations derived in this section is henceforth denoted as *analytical I* method.

#### 2.3.2. Spike response model formulation of the BCPNN model

As starting point for a second event-driven analytical solution with less operations, we make use of the linearity of the BCPNN differential equations and formulate the dynamics as a spike response model, in accordance with the work of Gerstner and Kistler (2002). The presynaptic traces can be written as a response to spike times *t^f^_i_*:

(15) $$Z_{i}(t)=\sum_{t_{i}^{f}}\zeta_{i}(t-t_{i}^{\;f}),\;\zeta_{i}(t)={\text{e}}^{-\frac{t}{\tau_{z_{i}}}}\Theta (t)$$

(16) $$E_{i}(t)\text{​ }=\sum_{t_{i}^{f}}\alpha_{i}(t-t_{i}^{\;f}),\;\alpha_{i}(t)\text{​}=\text{​}a_{i}\left({\text{e}}^{-\frac{t}{\tau_{z_{i}}}}-{\text{e}}^{-\frac{t}{\tau_{e}}}\right)\Theta (t)$$

(17) $$\begin{array}{l} P_{i}(t)=\sum_{t_{i}^{f}}\pi_{i}(t-t_{i}^{\;f}),\;\pi_{i}(t)=a_{i}[b_{i}\left({\text{e}}^{-\frac{t}{\tau_{z_{i}}}}-{\text{e}}^{-\frac{t}{\tau_{p}^{*}}}\right)\\ \;+c\left({\text{e}}^{-\frac{t}{\tau_{p}^{*}}}-{\text{e}}^{-\frac{t}{\tau_{e}}}\right)]\Theta (t). \end{array}$$

**Figure 3:**
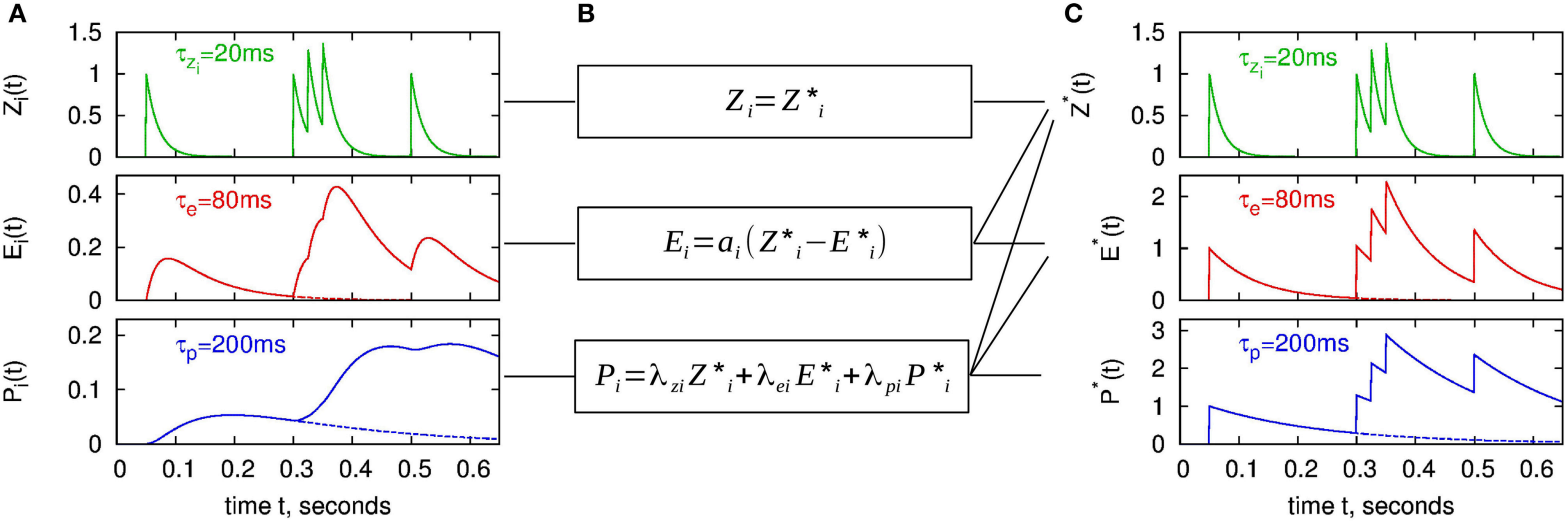
**BCPNN dynamics in two different representations for an example spike train. (A)** Presynaptic traces according to the original model formulation (*Z_i_*, *E_i_*, *P_i_*), as expressed in Equations (7–9). **(C)** “Exponential” traces Z^*^_i_, E^*^_i_, P^*^_i_ used for the analytical II solution with minimal number of calculations, according to Equations (32–34). Dashed lines in **A** and **C** denote spike response kernels, i.e., responses to a single spike. **(B)** Transformation between the two different representations (Equations 29, 30, 46).

Here Θ(·) denotes the Heaviside step function. ζ_*i*_, α_*i*_, and π_*i*_ are the spike response kernels for the *Z_i_*, *E_i_* and *P_i_* traces. One can obtain Equations (15–17) from the analytical solution by setting *Z_i_*(*t*^last^) = 1, *E_i_*(*t*^last^) = 0, *P_i_*(*t*^last^) = 0 in Equations (7–9). The spike response kernels ζ_*i*_, α_*i*_, and π_*i*_ are shown in the left panel of Figure 3 as dashed lines. The postsynaptic traces can be analogously formulated, by replacing *i* with *j* in Equations (15–17).

For the synaptic trace variables *E_ij_* and *P_ij_* the spike response formulation becomes more sophisticated: Therefore, we consider the product *Z_i_Z_j_*, which after inserting the spike response formulation of *Z_i_* and *Z_j_* is given by:

(18) $$Z_{i}Z_{j}=\sum_{t_{i}^{f}}\zeta_{i}(t-t_{i}^{\;f})\cdot \sum_{t_{j}^{f}}\zeta_{j}(t-t_{j}^{\;f})$$

(19) $$=\sum_{t_{i}^{f}}\sum_{t_{j}^{f}}{\text{e}}^{-\frac{t\;-\;t_{i}^{f}}{\tau_{z_{i}}}}{\text{e}}^{-\frac{t\;-\;t_{j}^{f}}{\tau_{z_{j}}}}\Theta (t-t_{i}^{\;f})\Theta (t-t_{j}^{\;f})$$

(20) $$\begin{array}{l} =\sum_{t_{i}^{f}}Z_{j}(t_{i}^{\;f}){\text{e}}^{-(t\;-\;t_{i}^{f})(\frac{1}{\tau_{z_{i}}}\;+\;\frac{1}{\tau_{z_{j}}})}\Theta (t-t_{i}^{\;f})\\ +\sum_{t_{j}^{f}}Z_{i}(t_{j}^{\;f}){\text{e}}^{-(t\;-\;t_{j}^{f})(\frac{1}{\tau_{z_{i}}}\;+\;\frac{1}{\tau_{z_{j}}})}\Theta (t-t_{j}^{\;f})\;. \end{array}$$

For Equation (20) we employed the fact that for the spike response of a presynaptic spike at time *t^f^_i_*, we can neglect the contribution of future postsynaptic spikes with *t^f^_j_* > *t^f^_i_*, and vice versa. Hence, similar to the *Z_i_* and *Z_j_*, the product *Z_i_Z_j_* can be written by means of the spike kernels ζ_*ij*_:

(21) $$\begin{array}{l} Z_{i}Z_{j}=\sum_{t_{i}^{f}}Z_{j}(t_{i}^{f})\zeta_{ij}(t-t_{i}^{f})+\sum_{t_{j}^{f}}Z_{i}(t_{j}^{f})\zeta_{ij}(t-t_{j}^{f}),\\ \zeta_{ij}(t)={\text{e}}^{-\frac{t}{\tau_{z_{ij}}}}\Theta (t). \end{array}$$

In contrast to *Z_i_* and *Z_j_*, where all spikes have equal strength, for *Z_i_Z_j_* the spike response of each presynaptic spike *t^f^_i_* is scaled by the current value of the postsynaptic *Z_j_* trace, respectively by *Z_i_*(*t^f^_j_*) for each postsynaptic spike *t^f^_j_*.

As the *E_ij_* trace is just a low-pass filtered version of the product *Z_i_Z_j_*, we can analogously write the *E_ij_* and *P_ij_* traces as spike response models:

(22) $$E_{ij}(t)=\sum_{t_{i}^{f}}Z_{j}(t_{i}^{f})\alpha_{ij}(t-t_{i}^{f})+\sum_{t_{j}^{f}}Z_{i}(t_{j}^{f})\alpha_{ij}(t-t_{j}^{f})$$

(23) $$P_{ij}(t)=\sum_{t_{i}^{f}}Z_{j}(t_{i}^{f})\pi_{ij}(t-t_{i}^{f})+\sum_{t_{j}^{f}}Z_{i}(t_{j}^{f})\pi_{ij}(t-t_{j}^{f}),$$

with spike response kernels:

(24) $$\alpha_{ij}(t)=a_{ij}\left({\text{e}}^{-\frac{t}{\tau_{z_{ij}}}}-{\text{e}}^{-\frac{t}{\tau_{e}}}\right)\Theta (t)$$

(25) $$\pi_{ij}(t)=a_{ij}[b_{ij}\left({\text{e}}^{-\frac{t}{\tau_{z_{ij}}}}-{\text{e}}^{-\frac{t}{\tau_{p}^{*}}}\right)+c\left({\text{e}}^{-\frac{t}{\tau_{p}^{*}}}-{\text{e}}^{-\frac{t}{\tau_{e}}}\right)]\Theta (t)$$

We remark that Equations (20, 22, 23) are ambiguous for the limit case of simultaneous pre- and postsynaptic spikes (*t^f^_i_* = *t^f^_j_*), as it is unclear whether the sampled *Z_i_* and *Z_j_* correspond to the values directly before or after the spikes. This is resolved in Section 2.3.3.

#### 2.3.3. BCPNN solution with reduced operations: analytical II

For the analytical update of the *Z*, *E* and *P* traces derived in Section 2.3.1, we observe that especially the update of *P* traces is expensive in terms of number of operations. In the presented BCPNN architecture, each MCU has approximately 10,000 inputs and correspondingly as many outputs. It would therefore be of great benefit to reduce the computational cost of the update of the synaptic traces. We achieve this by transforming the BCPNN variables to a new set of state variables that all decay exponentially over time and are only increased when a spike occurs. This is motivated by the spike response model formulation (Section 2.3.2), where the *Z_i_*, *E_i_*, *P_i_* traces are superpositions of the spike response kernels ζ_*i*_, α_*i*_, and π_*i*_, which in turn are linear combinations of the exponential functions ${\text{e}}^{-\frac{t}{\tau_{z_{i}}}},{\text{e}}^{-\frac{t}{\tau_{e}}}$, and ${\text{e}}^{-\frac{t}{\tau_{p}^{*}}}$. Due to the linearity of the system we can choose these exponentials as new state variables to equally describe the BCPNN dynamics.

This second analytic solution of the BCPNN model is henceforth called *analytical II* in this paper.

***2.3.3.1. Presynaptic traces***. For the presynaptic side, we introduce the new state variables Z^*^_i_, E^*^_i_, and P^*^_i_:

(26) $$Z_{i}^{*}(t)=\sum_{t_{i}^{f}}{\text{e}}^{-\frac{t-t_{i}^{f}}{\tau_{z_{i}}}}\Theta (t-t_{i}^{f})$$

(27) $$E_{i}^{*}(t)=\sum_{t_{i}^{f}}{\text{e}}^{-\frac{t-t_{i}^{f}}{\tau_{e}}}\Theta (t-t_{i}^{f})$$

(28) $$P_{i}^{*}(t)=\sum_{t_{i}^{f}}{\text{e}}^{-\frac{t-t_{i}^{f}}{\tau_{p}^{*}}}\Theta (t-t_{i}^{f})\;,$$

which can be used to express *Z_i_*, *E_i_*, and *P_i_*:

(29) $$Z_{i}(t)=Z_{i}^{*}(t)$$

(30) $$E_{i}(t)=a_{i}\left(Z_{i}^{*}(t)-E_{i}^{*}(t)\right)$$

(31) $$P_{i}(t)=a_{i}\left[b_{i}\left(Z_{i}^{*}(t)-P_{i}^{*}(t)\right)+c\left(P_{i}^{*}(t)-E_{i}^{*}(t)\right)\right]\;.$$

The time course of the new state variables as a response to an example spike train is shown in Figure 3C. Note that we have introduced Z^*^_i_ although it is identical to *Z_i_* in order to emphasize the concept of the new representation with exponentially decaying state variables.

Instead of performing an event-based update of the original state variables *Z_i_*, *E_i_*, and *P_i_*, we can update Z^*^_i_, E^*^_i_, and P^*^_i_: Given that there is no spike between *t*^last^ and *t*, the state evolves from *t*^last^ to *t*, with Δ*t* = *t* − *t*^last^, as:

(32) $$Z_{i}^{*}(t)=Z_{i}^{*}(t^{\text{last}})\cdot {\text{e}}^{-\frac{\Delta t}{\tau_{z_{i}}}}+S_{i}(t)$$

(33) $$E_{i}^{*}(t)=E_{i}^{*}(t^{\text{last}})\cdot {\text{e}}^{-\frac{\Delta t}{\tau_{e}}}+S_{i}(t)$$

(34) $$P_{i}^{*}(t)=P_{i}^{*}(t^{\text{last}})\cdot {\text{e}}^{-\frac{\Delta t}{\tau_{p}^{*}}}+S_{i}(t)$$

Thus, between any two times we only have to calculate the exponential decay with τ*_z_i__*, τ_*e*_, and τ^*^_p_. At a new spike, we add 1 to all of the new state variables, compared to the classical lazy model, where only *Z_i_* is increased (cf. Figure 3). Of course, equivalent new state variables and the same updating scheme can be used for the postsynapic side.

***2.3.3.2. Synaptic traces***. For updating the synaptic variables, an analogy can be made to the presynaptic traces. Again, we introduce new state variables E^*^_ij_ and P^*^_ij_:

(35) $$E_{ij}^{*}(t)\text{​ }=\sum_{t_{i}^{f}}Z_{j}(\text{​}t_{i}^{f}\text{​}){\text{e}}^{-\frac{t-t_{i}^{f}}{\tau_{e}}}\Theta (t\text{​}-\text{​}t_{i}^{f})\text{​}+\text{​}\sum_{t_{j}^{f}}Z_{i}(\text{​}t_{j}^{f}\text{​}){\text{e}}^{-\frac{t-t_{j}^{f}}{\tau_{e}}}\Theta (\text{​}t\text{​}-\text{​}t_{j}^{f}\text{​})\;$$

(36) $$P_{ij}^{*}(t)\text{ ​}=\sum_{t_{i}^{f}}Z_{j}(\text{​}t_{i}^{f}\text{​}){\text{e}}^{-\frac{t-t_{i}^{f}}{\tau_{p}^{*}}}\Theta (t\text{​}-\text{​}t_{i}^{f})\text{​}+\text{​}\sum_{t_{j}^{f}}Z_{i}(\text{​}t_{j}^{f}\text{​}){\text{e}}^{-\frac{t-t_{j}^{f}}{\tau_{p}^{*}}}\Theta (\text{​}t\text{​}-\text{​}t_{j}^{f}\text{​})\;$$

These, together with Z^*^_i_ and Z^*^_j_, can be used to express *E_ij_* and *P_ij_*:

(37) $$E_{ij}(t)=a_{ij}\left(Z_{i}^{*}(t)Z_{j}^{*}(t)-E_{ij}^{*}(t)\right)$$

(38) $$P_{ij}(t)\text{ ​}=a_{ij}\left[b_{ij}\left(Z_{i}^{*}(t)Z_{j}^{*}(t)\text{​}-\text{​}P_{ij}^{*}(t)\right)\text{​}+c\left(P_{ij}^{*}(t)\text{​}-\text{​}E_{ij}^{*}(t)\right)\right]\;\;\;\;$$

We first consider the event-based update of the new synaptic state variables E^*^_ij_ and P^*^_ij_ for a presynaptic spike only (which is equivalent to a postsynaptic spike only). The case of simultaneous pre- and postsynaptic spikes is treated separately afterwards. In order to advance E^*^_ij_ and P^*^_ij_ from their last updated time *t*^last^ to *t*, with Δ*t* = *t* − *t*^last^ and no spike within this interval, the update goes as follow:

(39) $$E_{ij}^{*}(t)=E_{ij}^{*}(t^{\text{last}})\cdot {\text{e}}^{-\frac{\Delta t}{\tau_{e}}}+S_{i}(t)\cdot Z_{j}(t)$$

(40) $$P_{ij}^{*}(t)=P_{ij}^{*}(t^{\text{last}})\cdot {\text{e}}^{-\frac{\Delta t}{\tau_{p}^{*}}}+S_{i}(t)\cdot Z_{j}(t),$$

i.e., E^*^_ij_ and P^*^_ij_ decay exponentially from their last states and, for the case of a presynaptic spike *t^f^_i_* at time *t*, increase by the sampled postsynaptic *Z_j_*(*t*) trace. Here lies the difference to the presynaptic update, where each spike has the same effect, whereas the synaptic E^*^_ij_ and P^*^_ij_ traces are increased depending on the current *Z_j_* value of the postsynaptic side, as the synaptic traces keep track of the *overlap* of pre- and postsynaptic activity.

The case of concurrent pre- and postsynaptic spikes is not well defined in the formulas for E^*^_ij_ and P^*^_ij_ (Equations 35, 36) and in the spike response model formulation (Equations 22, 23). Therefore, we turn back to the product *Z_i_Z_j_*, which at simultaneous pre- and postsynaptic spikes is increased by

(41) $$\Delta_{ij}=Z_{i}^{+}Z_{j}^{+}-Z_{i}^{-}Z_{j}^{-}\;.$$

Here *Z*^−^_*i*_ (*Z*^−^_*j*_) denotes the Z-trace before the evaluation of a presynaptic (postsynaptic) spike, and *Z*^+^_*i*_ (*Z*^+^_*j*_) after the evaluation:

(42) $$Z_{i}^{+}=Z_{i}^{-}+S_{i}\;,\;Z_{j}^{+}=Z_{j}^{-}+S_{j}\;,$$

where *S_i_* (*S_j_*) is only non-zero if there is a presynaptic (postsynaptic) spike at the current time. Inserting Equation (42) into Equation (41) yields

(43) $$\Delta_{ij}=(Z_{i}^{-}+S_{i})(Z_{j}^{-}+S_{j})-Z_{i}^{-}Z_{j}^{-}$$

(44) $$\;=S_{i}Z_{j}^{-}+S_{j}Z_{i}^{-}+S_{i}S_{j}$$

(45) $$\;=S_{i}Z_{j}^{-}+S_{j}Z_{i}^{+}\;.$$

The increment Δ_*ij*_ not only describes the change of *Z_i_Z_j_*, but also applies to updates for the new synaptic traces E^*^_ij_ and P^*^_ij_. Equation (44) can be used when both spikes are evaluated *synchronously*, Equation (45) when both spikes are evaluated *consecutively*, i.e., when first the presynaptic spike is processed (first summand), and afterwards the postsynaptic spike (second summand). For the event-based benchmark simulations (Sections 2.2.2 and 3.1.2), where all spikes are discretized to multiples of *dt*, the latter strategy for Δ_*ij*_ is used for the update in the synapse array: first all presynaptic spikes are evaluated, then all postsynaptic spikes.

***2.3.3.3. Initialization of exponential state variables***. This section explains how to set the initial values of the new state variables (Z^*^, E^*^, P^*^) from a given set of *Z*, *E*, *P* traces. Therefore, we first shorten the transformation formula of *P_i_* (Equation 31) with new coefficients as:

(46) $$P_{i}=\lambda_{zi}Z_{i}^{*}+\lambda_{ei}E_{i}^{*}+\lambda_{pi}P_{i}^{*}$$

(47) $$\lambda_{zi}=a_{i}b_{i}\;,\;\lambda_{ei}=-a_{i}c\;,\;\lambda_{pi}=a_{i}\left(c-b_{i}\right)\;.$$

For brevity, we have left out the time dependence of the states. The equivalent simplification can be applied for the postsynaptic traces. Similarly, the synaptic traces (Equations 37, 38) can be written as

(48) $$E_{ij}=a_{ij}\left(Z_{i}^{*}Z_{j}^{*}-E_{ij}^{*}\right)$$

(49) $$P_{ij}=\lambda_{zij}Z_{i}^{*}Z_{j}^{*}+\lambda_{eij}E_{ij}^{*}+\lambda_{pij}P_{ij}^{*}\;,$$

with coefficients

(50) $$\lambda_{zij}=a_{ij}b_{ij}\;,\;\lambda_{eij}=-a_{ij}c\;,\;\lambda_{pij}=a_{ij}\left(c-b_{ij}\right)\;.$$

To turn the set of *Z*, *E*, *P* variables into the new state variables (Z^*^, E^*^, P^*^), the following reverse transformation holds:

(51) $$Z_{i}^{*}=Z_{i}$$

(52) $$E_{i}^{*}=-\frac{E_{i}}{a_{i}}+Z_{i}^{*}$$

(53) $$P_{i}^{*}=\frac{1}{\lambda_{pi}}\left(P_{i}-\lambda_{zi}Z_{i}^{*}-\lambda_{ei}E_{i}^{*}\right)$$

Note that the transformation has to be performed in the above order. The synaptic values are set as follows:

(54) $$E_{ij}^{*}=-\frac{E_{ij}}{a_{ij}}+Z_{i}^{*}Z_{j}^{*}$$

(55) $$P_{ij}^{*}=\frac{1}{\lambda_{pij}}\left(P_{ij}-\lambda_{zij}Z_{i}^{*}Z_{j}^{*}-\lambda_{eij}E_{ij}^{*}\right)$$

### 2.4. Benchmarks

To validate our implementation of the BCPNN we used several benchmarks, targeting either simulation run time or accuracy. As infrastructure for the simulations we used a cluster with Intel® Xeon® CPU E5-2690 2.90 GHZ. All benchmarks were implemented in C++ and compiled with GCC 4.7.1. All simulations were single-threaded. The time constants and other BCPNN parameters used for the benchmarks are listed in Table 1.

**Table 1:** **Parameters used in the execution time and accuracy benchmarks**.

**Synapse model**	
Parameters	τ*_z_i__* = 10 ms presynaptic *Z* trace time constant
	τ*_z_j__* = 15 ms postsynaptic *Z* trace time constant
	τ_*e*_ = 20ms *E* trace time constant
	τ_*p*_ = 1000 ms *P* trace time constant
	κ = 1 learning rate
	ϵ = 0.001 minimum activity

*Note that the values represent only one possible parameter set. Plausible ranges for the time constants are given in the text (Section 2.1.1). The execution time is not affected by the choice of the parameters, but, of course, the accuracy results may change when using different parameters*.

#### 2.4.1. Simulation run time

To compare the computational cost of the different update strategies, we simulated the synaptic dynamics of a full hypercolumn with 10,000 inputs and 100 MCUs, see Figure 2. For both pre- and postsynaptic units we use independent Poisson spike trains, which are pre-generated and then read from a file to the main program, so that equal spike trains are used for the different update strategies. The simulation runs for 10 s, the Poisson rate is swept over a range of 0.01–100 Hz. For each rate and update strategy we assess the execution time per simulated second as the average of 5 runs with different random seeds. Although independent Poisson spike trains for the pre- and postsynaptic units will not be the case in realistic BCPNN applications including learning and retrieval of patterns, they sufficiently model the probabilistic nature of the MCUs and are thus favorable compared to regular spike trains. In order to measure only the computational cost of the synaptic updates, the stochastic MCUs are not simulated in this benchmark. However, for a fair comparison of the update strategies, we calculate the support value *s_j_* (Equation 3) for all postsynaptic units at each time step, so that all β_*j*_ are calculated at every time step, and the weights *w_ij_* are computed whenever a spike of presynaptic unit *i* arrives.

#### 2.4.2. Accuracy comparison

As many published results are based on an explicit Euler method (see e.g., Johansson and Lansner, 2007; Berthet et al., 2012; Kaplan and Lansner, 2014), we compare this numerical method to an exact analytical one in Section 3.2.2. Furthermore, we investigate the influence of using fixed-point operands with different number of bits instead of floating point numbers with double precision. For this purpose, we implemented a single BCPNN synapse in C++ with templates allowing the comparison of different number formats, making use of an in-house developed fixed-point library.

As stimuli for the BCPNN synapse we generated pre- and postsynaptic spike trains according to a homogeneous Poisson process with rate *r*. For the accuracy benchmarks not only the update frequency is important but also that different dynamical ranges of the BCPNN variables can be triggered, which requires different levels of correlation. To achieve that, we follow Kuhn et al. (2003) and create pre- and postsynaptic Poisson spike trains that share a fraction of *c* correlated spike times. Therefore, we create one correlated Poisson spike train with rate *c* · *r*, and two independent Poisson spike trains with rate (1 − *c*) · *r* for the pre- and postsynaptic side. The correlated spike times are then added to both independent spike trains. To avoid a systematic zero-lag between pre- and postsynaptic spike times, the correlated spike times of the postsynaptic side are jittered according to a Gaussian distribution with standard deviation σ = 5 ms.

We run multiple simulations to investigate the effects of the Euler method and fixed-point operands, respectively. For each accuracy setting, stimuli are generated using 11 correlation factors *c* ranging from 0 to 1 in intervals of 0.1. For each of the different correlation factors, 10 different seeds are used for the Poisson processes, resulting in 110 simulations per accuracy setting. The stimuli are generated with an average rate of 1 Hz and the duration of each simulation is 1000 s. For the Euler method, spike times are set to multiples of the time step to avoid time discretization errors (Henker et al., 2012). For fixed-point operands, spike times are generated with a resolution of 0.01 ms.

To assess the accuracy of the different implementations, we consider absolute errors *e*_abs_:

(56) $$e_{\text{abs}}\;=|x-\hat{x}|\;,$$

where *x* denotes the exact value (analytical solution with floating point double precision) and $\hat{x}$ is the approximation (either the Euler solution or the analytical solution with fixed-point operands). By point wise comparing each of the state variables (e.g., *w_ij_*, *P_ij_* …), the accuracy can be assessed. The mean absolute error *e*_abs_ is the average of the single absolute errors determined at each time of a pre- or postsynaptic spike over all simulation runs. The normalized mean absolute error (NMAE) is the mean absolute error divided by the range of observed values *x*:

(57) $$\text{NMAE}=\frac{\bar{e_{\text{abs}}}}{x_{\text{max}}-x_{\text{min}}}\;,$$

which allows to compare the accuracy of several variables with different scales.

## 3. Results

The two analytic solutions for spike-based BCPNN derived in Section 2.3 allow an efficient event-driven simulation of BCPNNs. In Section 3.1 we investigate how this reduces the computational footprint of BCPNN learning both formally and empirically. Aiming also for a small memory footprint, we evaluate the use of fixed-point numbers for the storage of BCPNN state variables, and compare the introduced discretization errors with the errors caused by the fixed step size simulation with the Euler method (Section 3.2).

### 3.1. Comparison of simulation strategies

In this section we compare the computational efficiency of the two analytical solutions of the BCPNN equations against each other and to the commonly used fixed step size implementation with the Euler method. We also investigate the benefit of using look-up tables for exponential decays in the analytical II method.

#### 3.1.1. Number of operations

We start with a formal comparison between the two analytical update solutions by counting the steps of calculation required for an event-based update in each representation. Therefore, we categorize the operation into three classes: ADD combines both additions and subtractions, MUL stands for multiplications and divisions, EXP for calculations of the exponential function, and LOG for the natural logarithm.

Table 2 lists the number of operations needed by the analytical I and analytical II methods for different tasks: For the update of the presynaptic state variables (*Z_i_*, *E_i_*, *P_i_* resp. Z^*^_i_, E^*^_i_, P^*^_i_) at an incoming spike, most notably, the analytical II method requires 6 MUL and 3 ADD operations less than the analytical I method. Instead, when the *P_i_* value is retrieved, e.g., to calculate the synaptic weight *w_ij_*, the analytical I method requires zero operations, while the analytical II method requires 2 ADD and 3 MUL operations to calculate *P_i_* from Z^*^_i_, E^*^_i_, and P^*^_i_. Here, the difference between the two strategies manifests: while the analytical II is more efficient when the states are updated, it requires additional operations to determine the original states. Nevertheless, when adding up the counts of both tasks (pre-update and retrieval of *P_i_*), e.g., when β_*j*_ is updated after a postsynaptic spike, the analytical II is still much more efficient than the analytical I method.

**Table 2:** **Arithmetic operations per task for different analytical update methods**.

**Task**	**Operation**	**Analytical I**	**Analytical II**
Pre-update		Equations (7–9)	Equations (32–34)
	ADD	7	3
	MUL	12	6
	EXP	3	3
Retrieve *P_i_*			Equation (46)
	ADD	–	2
	MUL	–	3
Syn-update		Equations (12, 13)	Equations (39, 40)
	ADD	6	2
	MUL	13	5
	EXP	3	2
Retrieve *P_ij_*			Equation (49)
	ADD	–	2
	MUL	–	4
Update of *w_ij_*
at pre-spike	ADD	22	14
	MUL	39	29
	EXP	9	8
	LOG	1	1
Update of β_*j*_
at post-spike	ADD	8	6
	MUL	12	9
	EXP	3	3
	LOG	1	1

*ADD, additions and subtractions; MUL, multiplications and divisions; EXP, computations of exponential function; LOG, natural logarithm. The operation counts of the analytical I method correspond to optimized versions of the referenced formulas using pre-calculated coefficients and intermediate steps. The different tasks are further specified in the text*.

Similar results are found for the update of the synaptic state variables (*E_ij_*, *P_ij_* resp. E^*^_ij_, P^*^_ij_), where the advantage of the analytical II over the analytical I is even larger, cf. Table 2. Again, the analytical II strategy needs additional steps of computation for the retrieval of *P_ij_*. For the typical case of a presynaptic spike, where all traces and the weight are updated (task “update of *w_ij_* after pre-spike”) and which includes the retrieval of all *P*-traces, the analytical II requires considerably less operations than the analytical I method. Note that the speedup of the analytical II is even higher when processing a post-synaptic spike, as then the weight needs not be calculated and thus the *P* traces need not be retrieved.

If we consider an array of BCPNN synapses, as in a hypercolumn unit of the reduced cortex model (Figure 2), where the pre- and postsynaptic traces are handled at margins of the array, it is the update and retrieval of the *synaptic* BCPNN state variables that make up the majority of the calculations. Assuming equal mean firing rates for the pre- and postsynaptic units, the *P_ij_* values need to be retrieved on average only at every second spike event. In that case, the analytical II method requires roughly half the number of basic arithmetic operations (ADD and MUL) of the analytical I method, but only slightly less calculations of the natural exponential function.

#### 3.1.2. Simulation run time

**Figure 4:**
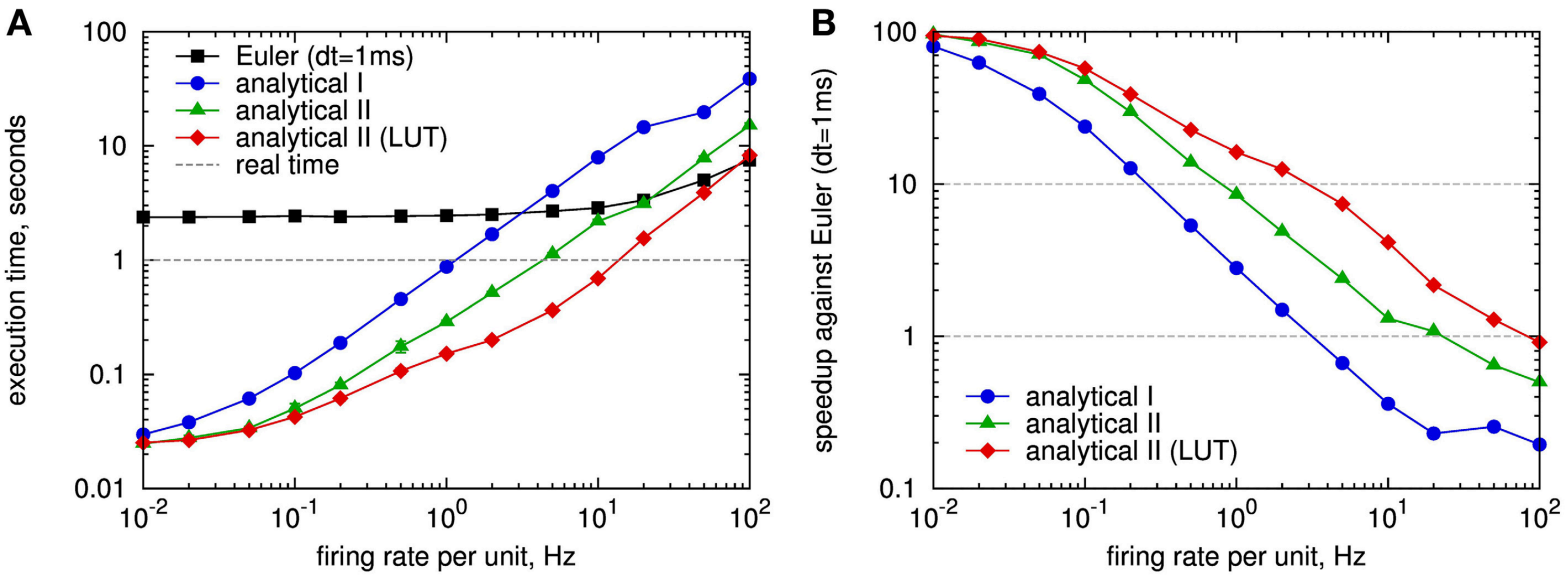
**Speed comparison of different simulation strategies for spike-based BCPNN:** fixed step size simulation with explicit Euler method with 1 ms time step (Euler, black curve), event-driven simulation with analytical update (analytical I, cf. Section 2.3.1, blue) and analytical update with exponential state variables (analytical II, cf. Section 2.3.3), with and without using look-up tables (LUTs) for the exponential function (red, resp. green). **(A)** Execution time for simulating a full hypercolumn unit with 1 million BCPNN synapses for 1 s with different Poisson firing rates applied to both pre- and postsynaptic units of the HCU (cf. Figure 2). **(B)** Speedup of event-based simulation methods with respect to the fixed step size simulation with Euler method in **A**. Look-up tables were implemented for the exponential decay of type $\exp(-\frac{\Delta t}{\tau })$ for the time constants τ*_z_i__*, τ*_z_j__*, τ_*e*_, τ^*^_p_. Each LUT had 3000 entries in steps of 1 ms.

As a complement to the formal comparison, we measured the simulation run time required to simulate the update of synapses of one HCU with 10,000 inputs and 100 outputs for the different update strategies. The results for a sweep over the Poisson firing rates of the inputs and outputs, which is described in detail in Section 2.4.1, are shown in Figure 4A. As expected, for the fixed step size simulation with explicit Euler method and *dt* = 1 ms the execution time depends only slightly on the spike frequency: It takes 2.4 s to simulate 1 s of the network for firing rates up to 10 Hz, only for higher rates the run time increases significantly, which can be attributed to the more frequent calculation of synaptic weights. In contrast, for the event-based methods the execution time strongly depends on the firing activity: For very low spike rates, there is a baseline computational cost that can be attributed to the calculation of all postsynaptic biases β_*j*_ and support values *s_j_* (Equation 3) at every time step (cf. Section 2.4.1). For Poisson rates of 0.1 Hz and higher, the execution time scales linearly with the firing rate. The update strategy with reduced operations (analytical II, green curve) clearly outperforms the conventional analytical update (analytical I, blue curve). For a typical average firing rate of 1 Hz of MCUs in a HCU (cf. Lansner et al., 2014) the analytical II strategy is more than 3 times faster than the real-time dynamics of the model, while the analytical I update runs approximately at real time. We remark that we optimized the C++ code of the analytical II update as good as possible, while the analytical I code is not optimized to the end. Thus, the results of latter can not be taken as final and should rather be interpreted as an intermediary result.

We compare the run time of the event-based methods directly to the fixed step size simulation in Figure 4B. For low spiking activity, the event-based methods are up to 100 times faster than the fixed step size method. At 1 Hz the analytical II strategy (green curve) runs more than 8 times faster than the simulation with Euler. Only for firing rates higher than 20 Hz the fixed step size approach is competitive with, respectively faster than the analytical II method.

Additional results for a 0.1 ms time discretization are provided in Appendix A4, showing a much higher speedup of event-driven methods against the fixed step size method.

#### 3.1.3. Look-up tables for exponential functions

In another simulation we investigated the benefit of using look-up tables (LUTs) for the exponential functions instead of computing the exponential at each event. This is motivated by the number of exponential decays calculated per update (cf. Table 2), as well as by a profiling of the implemented C++ program which shows that a huge amount of simulation time is spent in the computation of the exponential function. Look-up tables are especially beneficial in the used event-driven simulation (Section 2.2.2) where spike times are restricted to multiples of the time step *dt*. Calculations of the form

(58) $$LUT(N,\tau ,dt)={\text{e}}^{-\frac{N\cdot dt}{\tau }}$$

are performed very often, where *N* is the number of time steps that have elapsed since the last update, and τ is one of the four involved time constants τ*_z_i__*, τ*_z_j__*, τ_*e*_, τ^*^_p_. In a modified version of the analytical II implementation, we create look-up tables of Equation (58) for the four time constants, each with *L* entries for *N* = 1 … *L*. Only if the number of elapsed time steps between two updates is larger than *L*, the exponential function Equation (58) is computed on demand.

The results for using look-up tables in the analytical II method are included in Figure 4: The implementation with look-up tables (red curve) speeds up the simulation for Poisson rates starting from 0.1 Hz, and is up to 3 times faster than the version without LUTs at 10 Hz spiking activity. Here, the size of the LUTs was chosen as *L* = 3000, covering update intervals up to 3 s, so that for a Poisson rate of 1 Hz on average 95% of the inter spike intervals are handled by the look-up table. For the typical case of 1 Hz the LUT implementation is 1.9 times faster than the one without LUTs, 6.6 times faster than real time, and 16 times faster than the fixed step size simulation with explicit Euler method. For a wide spectrum of tested firing rates the analytical II solution with look-up tables is much more efficient than the fixed step size simulation with Euler, only for a firing rate of 100 Hz the latter performs slightly better (Figure 4B), so that in practical situations the fixed step size method becomes dispensable for the simulation of the abstract BCPNN cortex model.

### 3.2. Fixed-point numbers for BCPNN traces and their accuracy

To store all traces of the 1 million BCPNN synapses of a full HCU, one requires more than 12 MB assuming a single precision floating point number occupying 4 byte for each state variable (Lansner et al., 2014). Targeting an implementation of the BCPNN model on neuromorphic hardware, the use of fixed-point numbers can reduce the number of computational and storage resources, possibly at the price of loosing precision. Therefore, we investigate the accuracy of using fixed-point operands to store the state variables in the event-based simulation with analytical II method, and compare it to the accuracy of the fixed step size simulation with the Euler method.

#### 3.2.1. Value range estimation

For a fixed-point implementation of the BCPNN model, it is important to determine an upper bound of each state variable. This bound can be used to normalize the variables, so that an identical fixed-point representation can be used for all.

For a single exponential trace, be it the *Z_i_* and *Z_j_* traces in the standard analytical solution or the state variables of the analytical II solution, an upper bound can be calculated using a regular spike train with maximum rate *r*_max_. The value of this rate may be derived from the units' refractoriness period or as a multiple of the mean unit firing rate, accounting for short-term firing rate fluctuations. The upper bound can be calculated from the equilibrium state, where exponential decay and instantaneous increase at a spike equalize:

(59) $$\begin{array}{l} Z_{i}(t_{n})\overset{!}{=}Z_{i}(t_{n+1})=Z_{i}(t_{n})\cdot {\text{e}}^{-1/(r_{\text{max}}\cdot \tau_{z_{i}})}+S_{i}\\ \;\Rightarrow Z_{i,\text{max}}=\frac{S_{i}}{1-{\text{e}}^{-1/(r_{\text{max}}\cdot \tau_{z_{i}})}} \end{array}$$

**Figure 5:**
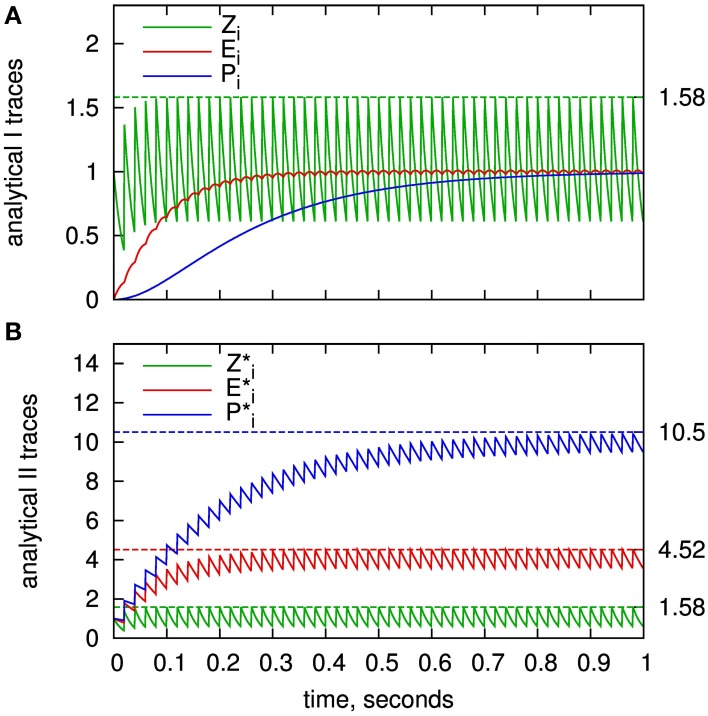
**Value range estimation of the BCPNN states variables:** Traces of the *Z*, *E*, and *P* states for a regular spike train with frequency of 50 HZ. **(A)** Presynaptic traces according to the standard analytical solution (analytical I), as expressed in Equations (7–9). **(B)** Exponential traces of the improved analytical solution analytical II, Equations (32–34). Analytically calculated limits according to Equation (59) are plotted as dashed lines. Time constants used here: τ*_z_i__* = 20 ms,τ_*e*_ = 80 ms and τ^*^_p_ = 200 ms.

The upper bounds of the presynaptic traces are illustrated in Figure 5A. They very closely match the actual maximums of the traces according to the employed regular spike train. For the traces of *E_i_* and *P_i_*, the same maximum as for *Z_i_* can be used in good approximation. This is motivated from the differential equations of the model given in Figure 1: The worst-case assumption for *Z_i_* from the maximum calculation would be a constant value of *Z*_*i*,max_. Given this input, the trace of *E_i_* would approach the same value. The same argument in turn holds for *P_i_*.

For the traces of the analytical II solution, Z^*^_i_, E^*^_i_, and P^*^_i_, Equation (59) can be used with according time constants. For *r*_max_ · τ ≫ 1, the maximum can be approximated as *r*_max_ · τ for an increment of *S_i_* = 1. The highest absolute value is reached for the longest time constant, which is τ_*p*_ = 1000 ms in our example parameter set. Assuming a refractoriness period of 1 ms, the worst-case upper bound would be P^*^_i,max_ ≈ 1000. For a fixed-point implementation, a width of 10 integer bits would be sufficient to avoid any unwanted saturation or overflows. It can be expected that the actual maximum of P^*^_i_ is significantly lower as it is extremely unlikely that a neuron (resp. a MCU) fires every 1 ms for a multitude of spikes. Thus, for a specific benchmark, a lower bound may be determined from simulation.

#### 3.2.2. Accuracy comparison

We ran multiple simulations to investigate the effects of the Euler method and fixed-point operands, respectively. For each accuracy setting, a single BCPNN synapse was stimulated by pre- and postsynaptic Poisson spike trains of 1 Hz average rate. We applied different levels of correlation between pre- and postsynaptic spike trains in order to generate wide value ranges of the BCPNN variables, especially for *w_ij_*. The simulation setup is described in detail in Section 2.4.2.

**Figure 6:**
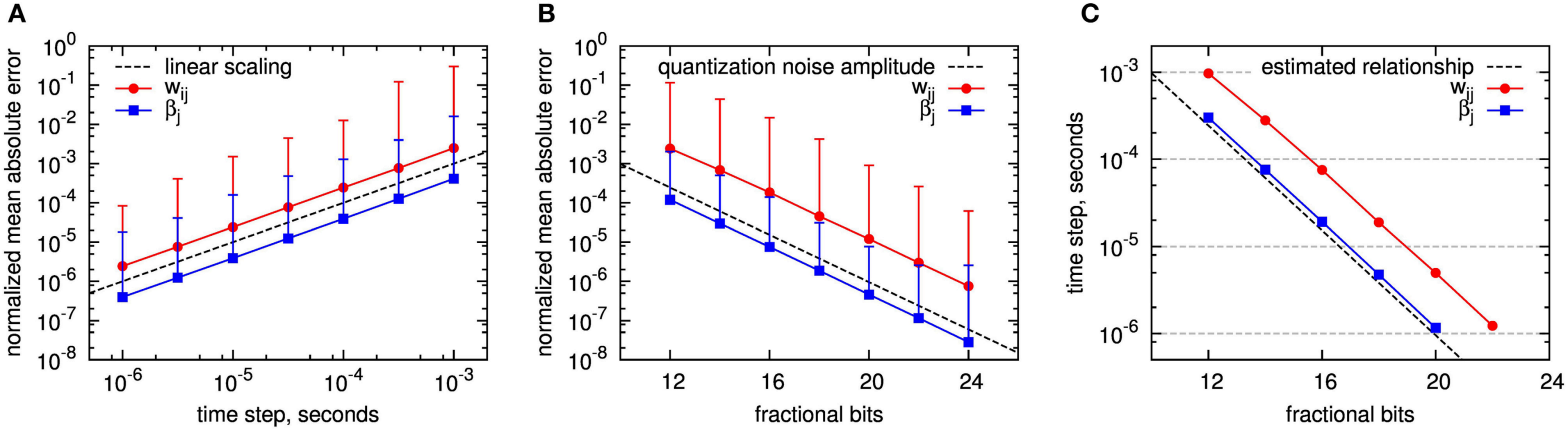
**Accuracy of fixed step size simulation with Euler method and event-driven analytic simulation using fixed point operands**. The accuracy of *w_ij_* and β_*j*_ is assessed by the normalized mean absolute error taken over a large set of experiments with the exact analytical solution as reference, see text for details. **(A)** Simulation with Euler, dependent on step size. The dashed line shows the linear scaling: *y*(*dt*) = *dt* · s^−1^. **(B)** Analytical solution with event-driven update (analytical II) using fixed-point representation with different counts of fractional bits. The dashed line shows the quantization noise amplitude: *y*(*b*) = 2^−*b*^. The error bars in **A** and **B** denote the normalized maximum absolute error recorded within all simulations per setup. **(C)** Comparison between the errors introduced by the Euler method and the use of fixed-point numbers with limited number of fractional bits: For *w_ij_* and β_*j*_ the location of *equal* mean absolute errors is plotted, depending on the step size for the Euler method, respectively the number of fractional bits of the fixed-point implementation. Dashed line: estimated relationship according to Equation (60).

The accuracy results for both Euler method and fixed-point operands are shown in Figure 6. As accuracy measure, we assess the normalized mean absolute error (NMAE) as described in Section 2.4.2. To get an impression of the variable ranges in the simulations, we give their average, minimum and maximum in Table 3. Note that we only show the errors for *w_ij_* and β_*j*_ (and not for the *Z*,*E*,*P* traces), as these are the only BCPNN variables that affect the activation of the postsynaptic units. As expected, the Euler method exhibits a linearly increasing accuracy with decreasing step size (Figure 6A). The accuracy is worse for the synaptic weight *w_ij_* than for the bias β_*j*_, as the *w_ij_* error is affected by the errors of *P_i_*,*P_j_*, and *P_ij_*, while β_*j*_ only depends on the accuracy of *P_j_*. For 1 ms step size, which we used for the execution time benchmarks, the normalized mean absolute error of the synaptic weight lies far below 1%. A reason for this relatively small error might be the exponentially decaying dynamics of the BCPNN variables, which keeps the accumulation of errors low.

**Table 3:** **Measured ranges of BCPNN state variables in accuracy simulations**.

**Variable**	**Mean**	**Min**	**Max**
*P_i_*	0.010	0.001	0.066
*P_j_*	0.015	0.001	0.097
*P_ij_*	0.0028	0.000	0.898
*w_ij_*	1.57	−6.75	5.35
β_*j*_	−4.38	−6.21	−2.32

For fixed-point operands, we used calculation with floating point precision, but quantized each intermediate result for a state variable to a fixed number of fractional bits. For the time constants and coefficients (Equations 47, 50) we used the highest available fixed-point precision (32 fractional bits) to minimize computational errors. This emulates the case that state variables are stored with limited precision to reduce storage space, but the arithmetic operations are designed such that they do not introduce additional numerical errors. Quantization errors can be modeled as a noise source with amplitude 2^−*b*^, where *b* is the number of fractional bits. All errors scale according to this noise source (compare dashed line in Figure 6B). Again, the accuracy is higher for β_*j*_ than for *w_ij_*, but now the ratio between *w_ij_* and β_*j*_ errors is larger than in the Euler simulation.

Comparing these results answers the question what fixed-point operand resolution is required in our optimized analytical solution to achieve at least the same accuracy as state-of-the-art Euler methods. This can be derived from curves with equal mean absolute error, as shown in the lower diagram of Figure 6C. In terms of scaling, Euler method and fixed-point operands compare as

(60) $$\bar{e_{\text{abs}}}=A_{\text{Euler}}\cdot dt=A_{\text{fixed}}\cdot 2^{-b}\;,$$

where *dt* is the step size of the Euler method and *A*_Euler_, *A*_fixed_ are variable-specific constants. The corresponding line *dt* = 2^−*b*^ is drawn as dashed line in the diagram. As expected from the previous results, the single errors follow this line, shifted by an offset. For a time step of *dt* = 0.1 ms 16 fractional bits or less are required to achieve at least the same accuracy in all variables. A number of integer bits is required in addition to represent values greater than one. As shown in Section 3.2.1, a maximum of 10 integer bits is required in a worst-case scenario for the employed parameter set.

For the simulation of the reduced modular model of the cortex described in Section 2.1.2, for which a 1 ms time step seems to provide sufficient results (Lansner et al., 2014), only 12 fractional bits, and thus at maximum 22 bits in total, are needed to ensure equal or better accuracy compared to using the Euler method. Hereby, the required memory per state variable decreases by almost one third compared to using single precision floating point numbers.

Considering a 0.1 ms time step and a 64 bit floating point representation, which is commonly used in state-of-the art neural network simulations, fixed-point numbers with less than 32 bits yield competitive accuracy, so that the memory footprint reduces even by more than a half.

## 4. Discussion

In this paper we derived two analytic solutions for the spike-based BCPNN learning rule. They enable an efficient event-driven simulation of spiking neural networks employing this learning rule, such as the reduced modular model of cortex (Lansner et al., 2014). The advantages of using an analytic over a fixed step size numeric solution are twofold: Firstly, it enables an event-driven update of the variables, and thereby significantly speeds up synaptic plasticity when interspike intervals are long compared to simulation time resolution. Secondly, it increases the precision of the calculations compared to fixed step size methods. Both aspects can be utilized for allocating resources in an existing hardware system efficiently or in conceiving a neuromorphic system based on the BCPNN computational paradigm.

### 4.1. Classification and limitations of optimization

In our simulations including 1 million BCPNN synapses with pre- and postsynaptic activity at 1 Hz, we were able to reduce the execution time by a factor of 16 compared to the conventional fixed step size simulation with explicit Euler. One hypercolumn unit of the reduced cortex model was simulated more than 6 times faster than real time on a single CPU. Several factors are responsible for that speedup:

By employing the analytical I solution of the BCPNN model, the *event-driven simulation* becomes feasible and clearly defeats the time-driven simulation at the chosen working point of 1 Hz firing rate. In general, the event-driven approach is mostly advantageous over the time-driven approach when the firing rates are low and connectivity is sparse (Brette et al., 2007). Hence, as long as the inter-spike intervals are large compared to the simulation step size, the analytic event-driven simulation can effectively reduce the execution time of spiking neural networks, independent whether the BCPNN synapses connect single neurons or more abstract units like in the cortex model.

The *analytical II* solution requires on average only half of the basic arithmetic operations of the conventional analytical I solution for an event-based update, and slightly less calculations of the exponential function. Here, the computational cost is reduced by representing the same BCPNN dynamics with a set of exponentially decaying state variables, which is possible due to the linearity of the system. A similar approach has been taken by Brette (2006) for the exact simulation of leaky integrate-and-fire neurons with synaptic conductances, albeit with the restriction of equal excitatory and inhibitory synaptic time constants. Quite the opposite, the only limitation for the BCPNN synapse model is that the decay time constants of the three low pass filtering stages must differ. Nevertheless, when a specific network model requires equal time constants, one can still switch to the analytical I solution provided in Appendix A2, or try slightly different parameters.

The usage of *look-up tables* for the frequent calculation of exponential decays can further accelerate the simulation by a factor of 2 or 3. Precalculated look-up tables are a common tool in event-driven neural network simulations to reduce the cost for the calculation of complex functions (Brette, 2006; Ros et al., 2006). For BCPNN, LUTs for the exponential decay are beneficial as long as the time constants are homogeneous and do not vary from synapse to synapse. In our hybrid simulation of a hypercolumn unit, where spikes are discretized to multiples of the simulation step size, look-up tables not only accelerate the simulation, but also provide the same accuracy as the solution without LUTs. For simulations with arbitrary update intervals, linear interpolation can be used to achieve almost exact results (Brette, 2006). Alternatively, for the case of the exponential function, the computation can be split into two steps, e.g., by first retrieving the EXP separately for the integer and fractional bits of the exponent, and then multiplying the two obtained results. There remains the question for the optimal size and resolution of the look-up tables, which must be chosen depending on the used hardware platform (available memory, cache size) and the inter spike interval distributions of actual network models.

The optimizations presented in this paper focus on reducing the computational footprint for the spike-based BCPNN learning rule: In our benchmarks we have considered either a single synapse or an array of synapses, but not the dynamics of neurons or the MCUs. The efficient simulation of large recurrent networks with many HCUs entails many new issues, e.g., the distribution of hypercolumns across compute nodes and memory, the communication of spikes between HCU or the buffering of spikes, and gives rise to separate studies that are clearly out of scope of this paper.

### 4.2. Accuracy

Fixed-point operands can reduce the memory footprint with the drawback of loosing precision compared to a floating point representation. To find the compromise between the two solutions, we assessed the accuracy of using fixed-point operands for the storage of the BCPNN state variables in an event-based simulation with the analytical II method (Section 3.2). The accuracy was compared to the errors introduced by the fixed step size simulation with explicit Euler method using 64 bit floating point numbers, which is commonly used in neural simulation. We found that fixed-point numbers with 22 bits assure equal or better accuracy for all BCPNN variables than the Euler method with 1 ms time step, resp. 26 bits for 0.1 ms time step.

The question remains about which accuracy is necessary in a practical situation. A previous study (Johansson and Lansner, 2004) on using fixed-point arithmetic for BCPNNs showed that an attractor network with 8 bit weights can offer the same storage capacity as an implementation with 32 bit floating point numbers. To achieve this, probabilistic fractional bits were used and the computation of the moving averages (low-pass filters) was performed in the logarithmic domain. Given these results, we speculate that also spike-based BCPNN can be implemented in fixed-point arithmetic with 16 or less bits without loosing computational capabilities, so that the required memory and memory bandwidth can be halved compared to 32 bit floating point numbers used in Lansner et al. (2014).

### 4.3. Neuromorphic hardware

Our optimizations can be directly incorporated for designing more efficient neuromorphic hardware systems. There are currently several diverse attempts for building large-scale hardware platforms, aiming for a more efficient simulation of large-scale neural models in terms of speed, power or scalability (Schemmel et al., 2012; Hasler and Marr, 2013; Benjamin et al., 2014; Furber et al., 2014; Merolla et al., 2014). As in our analysis, realizing synaptic plasticity is the most resource-demanding task, so that a focus of neuromorphic designs is in efficiently emulating plasticity mechanisms, most often implementing some variant of spike-timing dependent plasticity (STDP, Bi and Poo, 1998; Morrison et al., 2008) in analog or mixed-signal circuitry, see Azghadi et al. (2014) for a review.

An implementation of the BCPNN learning rule requires a stereotypical set of coupled low-pass filters, see Figure 1. Implementation of the rule in analog neuromorphic hardware is technically feasible, as there is large knowledge on building leaky integrators (Indiveri et al., 2011), and even the issue of long decay time constants in nanometer CMOS technologies can be resolved, e.g., with switched capacitor techniques (Noack et al., 2014). In this context, our optimized analytic solution offers an interesting alternative to the direct implementation of the original model equations: When using the analytical II solution, the stereotypical low-pass filters are only charged at incoming spikes, in contrast to the continuous coupling in a direct implementation. This alleviates the need for a continuous, variable-amplitude charging mechanism for the *E* and *P* traces. On the other hand, charging only at incoming spikes requires a more elaborate calculation of the output values, as present in the analytical II solution. However, this calculation needs to be performed only at spikes as well, allowing e.g., for an efficient implementation with switched-capacitor circuits.

The design of analog neuromorphic circuits is time-consuming and the circuits are affected by parameter variations due to device mismatch. Digital implementations are much less affected by these problems. They may be less energy and area efficient on the level of single elements and they do not allow for direct ion-channel-to-transistor analogies as employed in traditional neuromorphic designs (Hasler et al., 2007). However, they allow to fully utilize the energy efficiency and performance advantages of neural algorithms and modeling approaches, while offering better controllability and scalability.

Several purely digital neuromorphic systems support synaptic plasticity, implemented either on application-specific integrated circuits (Seo et al., 2011), on field-programmable gate arrays (FPGAs) (Cassidy et al., 2013) or a custom multiprocessor system using a larger number of general purpose ARM cores (SpiNNaker system, Furber et al., 2014). Recently Diehl and Cook (2014) showed how general STDP rules can be efficiently implemented on SpiNNaker, despite the system's restriction that synaptic weights can be modified only at the arrival of a presynaptic spike. By adopting their implementation of trace-based STDP, the event-driven spike-based BCPNN in variant analytical I or analytical II can be seamlessly integrated on the SpiNNaker hardware. As we do, Diehl and Cook (2014) use look-up tables for the exponential function; furthermore, SpiNNaker uses fixed-point arithmetic, so that our insights on the accuracy of fixed-point operands may find immediate application.

The event-driven approach is also amenable to state-of-the-art methods for reducing the energy of computation in digital systems. Recent multi-core hardware platforms support fine grained per-core power management, as for example demonstrated on the Tomahawk multiprocessor system-on-chip (MPSoC) architecture (Arnold et al., 2014; Noethen et al., 2014). By changing both the clock frequency and the core supply voltages of each processing element in a dynamic voltage and frequency scaling scheme (Höppner et al., 2012), the hardware performance can be adapted to the performance requirements to solve a particular part of the BCPNN in real time with reduced energy consumption, e.g., by regarding the number of incoming spikes per HCU per simulation step. In addition, within phases of low activity complete processing elements can be shut off to reduce leakage power consumption. Another candidate architecture for energy-efficient neural computation with BCPNNs is the multi-core Adapteva-Epiphany chip (Gwennup, 2011), which is optimized for power-efficient floating point calculations requiring only one fifth of the energy at equal flop rate as the state-of-the-art (but general-purpose) ARM's Cortex-A9 CPU.

Alternatively, spike-based BCPNN can be implemented on novel systems rather than on existing digital systems: For example, one may build dedicated digital hardware for the simulation of the BCPNN cortex model. Such a system containing compact supercomputer functionality can be prototyped in an FPGA with special units for the learning rule or the stochastic minicolumn units, and has therefore only low risk compared to mixed-signal implementations. Recently, Farahini et al. (2014) provided a concept for a scalable simulation machine of the abstract cortex-sized BCPNN model with an estimated power-dissipation of 6 kW in the technology of 2018, which is three orders of magnitudes smaller than for a full-cortex simulation on a supercomputer in comparable technology with 20 billion neurons and 10,000 times more synapses (see also Lansner et al., 2014). They assume the analytical I method for the event-driven updating of the BCPNN traces, and apply floating point units for arithmetic operations. Our work can further promote their performance: By using the analytical II method with look-up tables the computational cost can be further reduced; by moving to fixed-point arithmetics the required memory and memory bandwidth decreases, so that a low-power real-time simulation of the cortex becomes possible.

### 4.4. Outlook

Of course, our optimizations can also be used to boost the simulation of spike-based BCPNN on conventional computing systems. For example, already the supercomputer simulations of the reduced cortex model by Benjaminsson and Lansner (2011) showed weak scaling and achieved the real-time operation when simulating one HCU per processor with the fixed step size Euler method (*dt* = 1 ms) and spike-based BCPNN synapses without *E* traces (the *Z* traces are directly passed to the *P* traces). Such large-scale BCPNN simulations are mostly bounded by computation rather than by inter-process communication (Johansson and Lansner, 2007; Lansner et al., 2014), as the firing activity is low and the connectivity is sparse and patchy. Hence, we conjecture that with our approach a speedup factor of 10 or more might be achieved. At the same time, our results can accelerate the simulations of small or medium-scale neural networks employing the spike-based BCPNN learning rule, with applications ranging from olfaction modeling (Kaplan and Lansner, 2014), reward learning (Berthet et al., 2012) to probabilistic inference (Tully et al., 2014). Regardless of whether the BCPNN is implemented in neuromorphic hardware, on a single PC or on supercomputers, the presented optimization through event-driven simulation with look-up tables can boost the success of the BCPNN paradigm as a generic plasticity algorithm in neural computation.

Furthermore, the BCPNN abstraction constitutes an alternative approach to tackle the energy efficiency wall for brain-sized simulations discussed in Hasler and Marr (2013): Instead of simulating every single neuron and synapse, one can choose a higher level of abstraction for the basic computational units in the brain (e.g., a minicolumn), use a powerful learning rule (e.g., spike-based BCPNN), and implement such networks in a lazy simulation scheme (e.g., on dedicated digital hardware), to finally achieve a very energy-efficient simulation of the brain.

## Author contributions

Bernhard Vogginger, René Schüffny, Anders Lansner, Love Cederström, Johannes Partzsch, and Sebastian Höppner designed and conceived this work. René Schüffny and Bernhard Vogginger developed the analytical II solution. Bernhard Vogginger, Love Cederström, Johannes Partzsch, and Anders Lansner provided simulation or analysis code. Bernhard Vogginger, Love Cederström and Johannes Partzsch performed the experiments and analyzed the data. Bernhard Vogginger, René Schüffny, Anders Lansner, Love Cederström, Johannes Partzsch, and Sebastian Höppner wrote the paper and approved the final manuscript.

### Conflict of interest statement

The authors declare that the research was conducted in the absence of any commercial or financial relationships that could be construed as a potential conflict of interest.
