# Supplementary material for: Reducing the computational footprint for real-time BCPNN learning
Source: Front Neurosci. 2015 Jan 22;9:2. doi: 10.3389/fnins.2015.00002 (PMC4302947; doi:10.3389/fnins.2015.00002)
Supplement: Supplementary file 1 [file DataSheet1.PDF]

A APPENDIX

For completeness, this appendix covers the solutions of the analytical I method (cf. section 2.3.1) and the spike response model formulation (cf. section 2.3.2) for the cases where not all involved time constants are different. Furthermore, we include results of the execution time comparison of the different update strategies with a finer time discretization than in section 3.1.2.

A.1 SPIKE RESPONSE MODEL FORMULATION OF BCPNN MODEL FOR CASES WITH EQUAL TIME CONSTANTS

Spike response model (SRM) formulation of E_i trace with $\tau_{z_i} = \tau_e$:

$$\alpha_i(t) = \frac{t}{\tau_{z_i}} \mathrm{e}^{-\frac{t}{\tau_{z_i}}} \Theta(t) \tag{A1}$$

SRM formulation of P_i trace with $\tau_{z_i} = \tau_e$ and $\tau_p^* \neq \tau_{z_i}$:

$$\pi_i(t) = \frac{\tau_p^*}{\tau_{z_i}} b_i \left[\left(\frac{t}{\tau_p^*} - b_i \right) \mathrm{e}^{-\frac{t}{\tau_{z_i}}} + b_i \mathrm{e}^{-\frac{t}{\tau_p^*}} \right] \Theta(t) \tag{A2}$$

SRM formulation of P_i trace with $\tau_{z_i} = \tau_e = \tau_p^*$:

$$\pi_i(t) = \frac{t^2}{2\tau_{z_i}^2} \mathrm{e}^{-\frac{t}{\tau_{z_i}}} \Theta(t) \tag{A3}$$

SRM formulation of P_i trace with $\tau_{z_i} \neq \tau_e$ and $\tau_p^* = \tau_{z_i}$:

$$\pi_i(t) = a_i \left[\frac{t}{\tau_{z_i}} \mathrm{e}^{-\frac{t}{\tau_{z_i}}} + c \left(\mathrm{e}^{-\frac{t}{\tau_{z_i}}} - \mathrm{e}^{-\frac{t}{\tau_e}} \right) \right] \Theta(t) \tag{A4}$$

SRM formulation of P_i trace with $\tau_{z_i} \neq \tau_e$ and $\tau_p^* = \tau_e$:

$$\pi_i(t) = a_i \left[a_i \left(\mathrm{e}^{-\frac{t}{\tau_{z_i}}} - \mathrm{e}^{-\frac{t}{\tau_e}} \right) - \frac{t}{\tau_e} \mathrm{e}^{-\frac{t}{\tau_e}} \right] \Theta(t) \tag{A5}$$

A.2 ANALYTICAL UPDATE OF BCPNN MODEL FOR CASES WITH EQUAL TIME CONSTANTS

Analytical update of E_i trace with $\tau_{z_i} = \tau_e$:

$$E_i(t) = E_i(t^{\mathrm{last}}) \cdot \mathrm{e}^{-\frac{\Delta t}{\tau_{z_i}}} + Z_i(t^{\mathrm{last}}) \frac{\Delta t}{\tau_{z_i}} \mathrm{e}^{-\frac{\Delta t}{\tau_{z_i}}} \tag{A6}$$

Analytical update of P_i trace with $\tau_{z_i} = \tau_e$ and $\tau_p^* \neq \tau_{z_i}$:

$$P_i(t) = P_i(t^{\mathrm{last}}) \cdot \mathrm{e}^{-\frac{\Delta t}{\tau_p^*}} + E_i(t^{\mathrm{last}}) b_i \left(\mathrm{e}^{-\frac{\Delta t}{\tau_{z_i}}} - \mathrm{e}^{-\frac{\Delta t}{\tau_p^*}} \right) + Z_i(t^{\mathrm{last}}) \frac{\tau_p^*}{\tau_{z_i}} b_i \left[\left(\frac{\Delta t}{\tau_p^*} - b_i \right) \mathrm{e}^{-\frac{\Delta t}{\tau_{z_i}}} + b_i \mathrm{e}^{-\frac{\Delta t}{\tau_p^*}} \right] \tag{A7}$$

Analytical update of P_i trace with $\tau_{z_i} = \tau_e = \tau_p^*$:

$$\pi_i(t) = P_i(t^{\mathrm{last}}) \cdot \mathrm{e}^{-\frac{\Delta t}{\tau_{z_i}}} + E_i(t^{\mathrm{last}}) \frac{\Delta t}{\tau_{z_i}} \mathrm{e}^{-\frac{\Delta t}{\tau_{z_i}}} + Z_i(t^{\mathrm{last}}) \frac{\Delta t^2}{2\tau_{z_i}^2} \mathrm{e}^{-\frac{\Delta t}{\tau_{z_i}}} \tag{A8}$$

Analytical update of P_i trace with $\tau_{z_i} \neq \tau_e$ and $\tau_p^* = \tau_{z_i}$:

$$P_i(t) = P_i(t^{\mathrm{last}}) \cdot \mathrm{e}^{-\frac{\Delta t}{\tau_{z_i}}} + a_i \frac{\Delta t}{\tau_{z_i}} \mathrm{e}^{-\frac{\Delta t}{\tau_{z_i}}} Z_i(t^{\mathrm{last}}) + \left(E_i(t^{\mathrm{last}}) - a_i Z_i(t^{\mathrm{last}}) \right) c \left(\mathrm{e}^{-\frac{\Delta t}{\tau_e}} - \mathrm{e}^{-\frac{\Delta t}{\tau_{z_i}}} \right) \tag{A9}$$

Analytical update of P_i trace with $\tau_{z_i} \neq \tau_e$ and $\tau_p^* = \tau_e$:

$$P_i(t) = P_i(t^{\mathrm{last}}) \cdot \mathrm{e}^{-\frac{\Delta t}{\tau_e}} + (a_i)^2 \left(\mathrm{e}^{-\frac{\Delta t}{\tau_{z_i}}} - \mathrm{e}^{-\frac{\Delta t}{\tau_e}} \right) Z_i(t^{\mathrm{last}}) + \left(E_i(t^{\mathrm{last}}) - a_i Z_i(t^{\mathrm{last}}) \right) \frac{\Delta t}{\tau_e} \mathrm{e}^{-\frac{\Delta t}{\tau_e}} \tag{A10}$$

A.3 DISCRETE CHANGES OF LEARNING RATE κ

By changing the learning rate κ, one can control the speed of learning in BCPNN networks, e.g. to freeze the synaptic weights in an attractor network after learning for a subsequent retrieval phase, or for applying reward learning (**Berthet et al.**, 2012). In a fixed step size simulation, where all states correspond to the same global time, the change of the learning rate can be easily implemented by just modifying parameter κ at the right time step.

The case is more complicated for event-driven simulations, where the states correspond to the time of their last update, which usually differs from synapse to synapse. Hence, when the learning rate changes at a discrete time t from κ to κ' the following applies to any event-based simulation: First, all state variables have to be updated to the current time t. Then all coefficients that involve κ have to be re-computed according to κ', in particular τ_p^*. For $\kappa' > 0$ the event-based updating can then continue as before.

The analytical II method requires an additional step: As the P traces undergo a continuous evolution whenever κ changes, the P^* traces must be transformed before continuing with the new coefficients given by $\tau_p^{*\prime} = \frac{\tau_p}{\kappa'}$. Hence, the following relation must hold, cf. eq. (46):

$$\lambda_{zi} Z_i^* + \lambda_{ei} E_i^* + \lambda_{pi} P_i^* = \lambda'_{zi} Z_i^* + \lambda'_{ei} E_i^* + \lambda'_{pi} P_i^{*\prime} \quad , \tag{A11}$$

where the primed variables correspond to the representation with κ'. By solving eq. (A11), the new $P_i^{*\prime}$ is computed as:

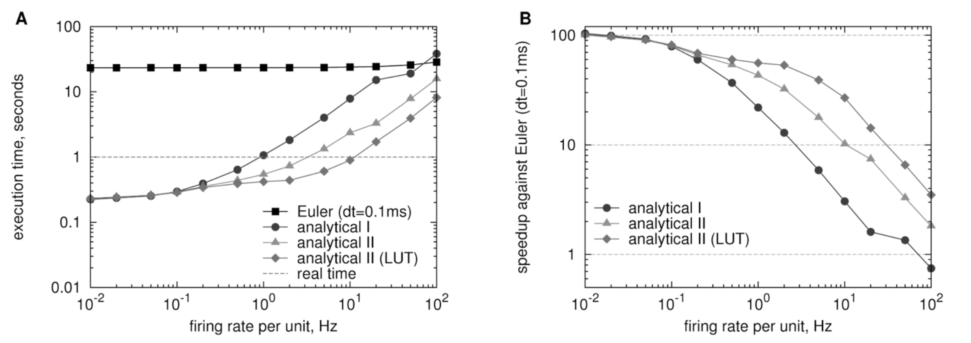

Figure A1. Speed comparison of different simulation strategies for spike-based BCPNN with 0.1 ms time discretization: fixed step size simulation with explicit Euler method with 0.1 ms time step (Euler, black curve), event-driven simulation with analytical update (analytical I, cf. section 2.3.1, blue) and analytical update with exponential state variables (analytical II, cf. section 2.3.3), with and without using look-up tables (LUTs) for the exponential function (red, resp. green). **(A)** Execution time for simulating a full hypercolumn unit with 1 million BCPNN synapses for one second with different Poisson firing rates applied to both pre- and postsynaptic units of the HCU (cf. Figure 2). **(B)** Speedup of event-based simulation methods with respect to the fixed step size simulation with Euler method in **A**. Look-up tables were implemented for the exponential decay of type $\exp(-\frac{\Delta t}{\tau})$ for the time constants $\tau_{z_i}, \tau_{z_j}, \tau_e, \tau_p^*$. Each LUT had 10 000 entries in steps of 0.1 ms.

$$P_i^{*\prime} = \frac{1}{\lambda'_{pi}} \left[(\lambda_{zi} - \lambda'_{zi}) Z_i^* + (\lambda_{ei} - \lambda'_{ei}) E_i^* + \lambda_{pi} P_i^* \right] \tag{A12}$$

Similarly, the new $P_{ij}^{*\prime}$ value is given from the old values of Z_i^*, Z_j^*, E_{ij}^* and P_{ij}^*:

$$P_{ij}^{*\prime} = \frac{1}{\lambda'_{pij}} \left[(\lambda_{zij} - \lambda'_{zij}) Z_i^* Z_j^* + (\lambda_{eij} - \lambda'_{eij}) E_{ij}^* + \lambda_{pij} P_{ij}^* \right] \tag{A13}$$

Special case $\kappa = 0$ When $\kappa = 0$, P_i^* is used to directly store the P_i value, such that the coefficients for the new state variables turn into $\lambda_{zi} = 0, \lambda_{ei} = 0, \lambda_{pi} = 1$. The same applies to the synaptic coefficients $\lambda_{zij} = 0, \lambda_{eij} = 0, \lambda_{pij} = 1$. The transformation of the P^* traces when κ switches between 0 and non-zero value again follows eqs. (A12) and (A13). Of course, while $\kappa = 0$, the P-traces, respectively the P^*-traces, remain constant and are not affected by any arriving spikes.

A.4 COMPARISON OF SIMULATION STRATEGIES WITH 0.1 MS TIME STEP

Figure A1 shows the same speed comparison measurements between the different update strategies as in Figure 4, but now using a 0.1 ms time discretization. This means that the fixed time step simulation with explicit Euler now runs with $dt = 0.1$ ms. Also the event-based simulations are affected, as the spike times are now distributed over a finer time grid. Furthermore, the calculation of the HCU state variables and the postsynaptic biases β_j is done in every time step and thus ten times more often (cf. section 2.4.1).

While the fixed step size simulation with explicit Euler is approximately ten times slower than with $dt = 1$ ms, the event-based methods are only barely affected from the finer discretization, e.g. the simulation of analytical II method at 1 Hz takes only twice as long as before, but is still faster than real time. The speedup compared to the Euler method (Figure A1**B**) however is much higher now.
